# A Prognostic Model for Critically Ill Children in Locations With Emerging Critical Care Capacity*

**DOI:** 10.1097/PCC.0000000000003394

**Published:** 2023-11-10

**Authors:** Arjun Chandna, Suy Keang, Meas Vorlark, Bran Sambou, Chhay Chhingsrean, Heav Sina, Pav Vichet, Kaajal Patel, Eang Habsreng, Arthur Riedel, Lazaro Mwandigha, Constantinos Koshiaris, Rafael Perera-Salazar, Paul Turner, Ngoun Chanpheaktra, Claudia Turner

**Affiliations:** 1 Cambodia Oxford Medical Research Unit, Angkor Hospital for Children, Siem Reap, Cambodia.; 2 Centre for Tropical Medicine & Global Health, University of Oxford, Oxford, United Kingdom.; 3 Department of Intensive Care Medicine, Angkor Hospital for Children, Siem Reap, Cambodia.; 4 Department of Global Child Health, Angkor Hospital for Children, Siem Reap, Cambodia.; 5 Department of Primary Care Health Sciences, University of Oxford, Oxford, United Kingdom.; 6 Angkor Hospital for Children, Siem Reap, Cambodia.

**Keywords:** critical care, low- and middle-income countries, pediatrics, risk, triage

## Abstract

**OBJECTIVES:**

To develop a clinical prediction model to risk stratify children admitted to PICUs in locations with limited resources, and compare performance of the model to nine existing pediatric severity scores.

**DESIGN:**

Retrospective, single-center, cohort study.

**SETTING:**

PICU of a pediatric hospital in Siem Reap, northern Cambodia.

**PATIENTS:**

Children between 28 days and 16 years old admitted nonelectively to the PICU.

**INTERVENTIONS:**

None.

**MEASUREMENTS AND MAIN RESULTS:**

Clinical and laboratory data recorded at the time of PICU admission were collected. The primary outcome was death during PICU admission. One thousand five hundred fifty consecutive nonelective PICU admissions were included, of which 97 died (6.3%). Most existing severity scores achieved comparable discrimination (area under the receiver operating characteristic curves [AUCs], 0.71–0.76) but only three scores demonstrated moderate diagnostic utility for triaging admissions into high- and low-risk groups (positive likelihood ratios [PLRs], 2.65–2.97 and negative likelihood ratios [NLRs], 0.40–0.46). The newly derived model outperformed all existing severity scores (AUC, 0.84; 95% CI, 0.80–0.88; *p* < 0.001). Using one particular threshold, the model classified 13.0% of admissions as high risk, among which probability of mortality was almost ten-fold greater than admissions triaged as low-risk (PLR, 5.75; 95% CI, 4.57–7.23 and NLR, 0.47; 95% CI, 0.37–0.59). Decision curve analyses indicated that the model would be superior to all existing severity scores and could provide utility across the range of clinically plausible decision thresholds.

**CONCLUSIONS:**

Existing pediatric severity scores have limited potential as risk stratification tools in resource-constrained PICUs. If validated, our prediction model would be a readily implementable mechanism to support triage of critically ill children at admission to PICU and could provide value across a variety of contexts where resource prioritization is important.

**RESEARCH IN CONTEXT**

- Capacity and demand are growing in settings with scarce resources for critical care and contextualized approaches are required to ensure sustainability.
- Generalizability of existing risk stratification tools to resource-constrained PICU contexts is uncertain.
- This study validated the performance of nine existing pediatric severity scores and developed a bespoke prognostic model for critically ill children admitted to a PICU in northern Cambodia.

**WHAT THIS STUDY MEANS**

- Existing pediatric severity scores show limited clinical utility in resource-constrained PICU settings.
- Including contextual determinants of clinical outcome can support holistic assessment of critical illness and improve risk stratification.
- If validated, the prognostic model developed in this study would be a readily implementable mechanism to support resource prioritization for critically ill children cared for in locations with emerging critical care capacities.

Historically, pediatric critical care has been perceived as too complex, expensive, or unethical to provide in settings where resources are scarce (1). These presumptions are countered by the fact that simple, low-cost interventions result in substantial improvements in health outcomes (2, 3). Consequently, capacities for pediatric critical care are growing in many resource-limited settings (2). However, need for critical care often outstrips supply (4). Resource stewardship is essential to promote sustainability of critical care services, especially in rural regions of many low- and middle-income countries (LMICs) (1, 2, 5).

Risk stratification tools can help target scarce resources optimally. However, tools developed in PICUs in high-income settings are time-consuming to compute and require diagnostic tests not routinely available in resource-constrained regions of LMICs (6). Furthermore, prognosis is influenced by the level of care available and underlying host susceptibility states, and hence adapted tools are required to support context-specific clinical decision-making (5, 7, 8). There have been calls to validate existing severity scores and develop new risk stratification tools for resource-constrained PICUs (5, 9). Unfortunately, most studies from LMIC PICUs are limited to urban centers, hampered by small sample sizes, and use methods incompatible with development of robust clinical severity scores or prediction models (10–14).

In this study, first, we externally validated nine existing severity scores using data from a PICU in northern Cambodia. Second, we developed a prognostic model, specifically to support risk stratification in resource-constrained PICU contexts.

## MATERIALS AND METHODS

This retrospective cohort study used consecutive admissions to the PICU at Angkor Hospital for Children (AHC), Siem Reap, Cambodia, between January 1, 2018, and January 1, 2020. Nonelective admissions of children older than 28 days old and younger than 16 years old were included. The study was approved by the AHC Research Committee (AHC 0656/20; October 2020), Cambodian National Ethics Committee for Health Research (NECHR 257; October 2020), and the Oxford Tropical Research and Ethics Committee (OxTREC 565-20; November 2020). Procedures followed were in accordance with the ethical standards of the responsible committees on human experimentation and with the Helsinki Declaration of 1975. The study is reported in accordance with the Transparent Reporting of a multivariable prediction model for Individual Prognosis Or Diagnosis guidelines (**Appendix 1**, http://links.lww.com/PCC/C445) (14).

### Study Setting

AHC is a pediatric healthcare organization with a nationwide catchment area. The hospital has 89 beds situated on two medical wards, a surgical ward, a special care baby unit, and neonatal and PICUs. The 14-bedded PICU has approximately 1000 annual admissions and is staffed by 30 nurses, four senior doctors, and five doctors in training. The level II unit provides mechanical and noninvasive ventilation (oxygen cylinders are delivered fortnightly), inotropic therapy, peritoneal dialysis, and specialist nursing (minimum 1:3 nurse-patient ratio) for critically ill children (15). A backup generator ensures continuity of electrical supply during infrequent power outages.

### Data Collection

PICU admissions were identified from the Hospital Information System and cross-checked against the unit’s admission logbook. Extracted data were recorded on a structured case report form (CRF). It was not possible to blind data collectors to patient outcome. The hospital admission and PICU vital sign proforma (**Appendix 2**, http://links.lww.com/PCC/C445) helped standardize data extraction. All variables were prospectively defined in a data dictionary to ensure consistency of interpretation across the research team. Each CRF was reviewed by a study physician (A.C. or S.K.) in consultation with the clinical records. Data were entered into an electronic database and 10% of CRFs were reviewed by a Data Manager (P.V.) to ensure a data entry error rate less than 0.5%.

### Severity Scores and Candidate Predictors

The results of two recent systematic reviews were supplemented by searching PubMed using synonyms of “pediatric” AND “severity score OR prediction model” (16, 17). Forty-nine scores or models were longlisted (**Appendix 3**, http://links.lww.com/PCC/C445). Nine severity scores were shortlisted for external validation (**Appendix 4**, http://links.lww.com/PCC/C445), including: Fluid Expansion as Supportive Therapy-Pediatric Emergency Triage (FEAST-PET) (18); Liverpool quick Sequential Organ Failure Assessment (LqSOFA) (19); Pediatric Advanced Warning Score (PAWS) (20); Pediatric Early Warning Score (PEWS) (21); the Irish PEWS (PEWS-IRISH); PEWS-Resource Limited (PEWS-RL) (22); quick Pediatric Logistic Organ Dysfunction-2 (qPELOD-2) (23); and the Systemic Inflammatory Response Syndrome (SIRS) (24). Neither the setting, population, nor outcome used for derivation were prerequisites for selection for external validation.

Baseline variables at PICU admission were extracted from the clinical records. Laboratory parameters measured within 24 hours of PICU admission were considered available at admission. A sensitivity analysis restricting this period to between 2 hours prior and up to 4 hours after admission was performed (**Appendix 5**, http://links.lww.com/PCC/C445) (25). For derivation of the new model, candidate predictors were selected a priori based on existing literature, expert knowledge, feasibility for implementation, and availability of data in the clinical records. Variables were selected across five domains (i.e., background, illness journey, cardiovascular, respiratory, and neurologic) to ensure inclusion of important contextual determinants of outcome often neglected by clinical risk scores developed in high-income settings. The 11 selected predictors were age, comorbidity status, weight-for-age *z* score, estimated travel time to hospital, route of admission to PICU, heart rate, capillary refill time, respiratory rate, peripheral oxygen saturation (Spo_2_), receipt of supplemental oxygen, and mental state.

### Outcomes

The primary outcome was death during PICU admission. Patients discharged from PICU to die at home were classified as meeting the primary outcome. A sensitivity analysis excluded these patients and those whose death was judged by either study physician (A.C. or S.K.) to have been related to a separate illness acquired during the PICU stay (Appendix 5, http://links.lww.com/PCC/C445).

The secondary outcome was death in the 12 months following PICU discharge. Caregivers of patients for whom postdischarge outcomes could not be determined from the clinical records were telephoned to ascertain vital status.

### Statistical Analyses

Routinely collected data indicated that 100 deaths were expected over 2 years, which would ensure sufficient outcome events for external validation of the existing severity scores (26). At this prevalence, and assuming a conservative Nagelkerke *R*^2^ of 0.15, up to 10 candidate predictors (events per parameter = 9.7) could be used to build the prediction model (R package: *pmsampsize*) (27, 28). In order to include interaction terms between age and each of heart rate and respiratory rate, penalization was used to shrink regression coefficients and permit inclusion of up to 13 parameters while minimizing the risk of overfitting.

Missing data were summarized (**Appendix 6**, http://links.lww.com/PCC/C445; R package: *naniar*) (29). Median imputation conditional on outcome status was proposed to address missingness. Sensitivity analyses comparing this to a full-case approach, as well as best- and worst-case imputation, produced similar results, confirming that median imputation was appropriate for the primary analysis.

Discrimination and calibration of each existing score was assessed by quantifying the area under the receiver operating characteristic curve (AUC; R package: *pROC*) (30) and plotting the proportion of admissions that met the primary outcome at each level of a score. Positive and negative likelihood ratios (PLRs and NLRs) were reported at each score’s cutoff to quantify the change in pre-test probability that a PICU admission would result in death. As a rule-of-thumb, a PLR greater than 10 or NLR less than 0.1 is deemed conclusive, a PLR between 5 and 10 or NLR between 0.1 and 0.2 is considered substantial, a PLR between 2 and 5 or NLR between 0.2 and 0.5 is regarded as small but important, and a PLR between 1 and 2 or NLR between 0.5 and 1 is likely clinically insignificant (31).

The relationship between continuous predictors and PICU survival status was examined using locally weighted scatterplot smoothing. Age-specific relationships for heart rate and respiratory rate were explored to account for known changes associated with physiologic maturation. Stratum-specific odds ratios and likelihood ratio tests (LRTs) were used to identify important interactions between age and each of heart rate and respiratory rate, as well as between Spo_2_ and receipt of supplemental oxygen. Penalized (ridge) logistic regression (penalty parameter = 0.026) was used to derive the model and shrink model coefficients to adjust for optimism (R package: *ridge*) (32). All predictors were prespecified and no predictor selection was performed during model development.

Discrimination (AUC), calibration (calibration intercept, slope, and plots), and classification indices (R package: *reportROC*) (33) were reported to summarize model performance. Recognizing that the relative value of a true positive (TP; PICU admission correctly identified as at high-risk of death) and false positive (FP; PICU admission incorrectly identified as at high-risk of death) will be context-dependent (e.g., depending on human and material capacities of a high-acuity area that at-risk PICU admissions might be triaged to), the clinical utility of the model was compared with the best-performing existing scores using decision curves to visualize their net-benefits over a range of clinically plausible decision thresholds (R package: *dcurves*) (34, 35).

All analyses were done in R, Version 4.2.2 (36).

## RESULTS

Between January 1, 2018, and January 1, 2020, there were 2066 PICU admissions, of which case notes were located for 2021 (97.8%). We included 1550 of 2021 admissions, giving an eligibility rate of 76.7% (**Appendix 7**, http://links.lww.com/PCC/C445). There were 1366 unique children, with 91.1% (1245/1366) admitted to the PICU only once during the study period. Median age at PICU admission was 14.0 months (interquartile range [IQR], 4.0–73.0 mo) and 59.8% of admissions (927/1550) were male (**Appendix 8**, http://links.lww.com/PCC/C445).

Admissions originated from 23 of Cambodia’s 25 provinces (**Appendix 9**, http://links.lww.com/PCC/C445). Median travel time was 69 minutes (IQR, 27–156 min). Over two-thirds of PICU admissions originated from the hospital’s emergency department (1067/1550; 68.8%), while 294 (294/1550; 19.0%) were intra-hospital transfers. All baseline severity scores were higher in admissions that resulted in death (*p* < 0.001) (**Appendices** 8 and **10**, http://links.lww.com/PCC/C445). The PICU mortality rate was 6.3% (97/1550) (**Appendix 11**, http://links.lww.com/PCC/C445, for causes of death). Median time to death was 4 days (IQR, 1–7 d) (**Appendix 12**, http://links.lww.com/PCC/C445). Outcomes 12 months post-PICU discharge were available for 782 of 1453 admissions (53.8%). Of these, 33 of 782 admissions (4.2%) resulted in death (25/672 [3.7%] individual children), a median of 1 month (IQR, 1–4 mo) postdischarge (Appendix 12, http://links.lww.com/PCC/C445).

### External Validation of Existing Severity Scores

All scores achieved comparable discrimination (AUCs, 0.71–0.76), except SIRS (AUC, 0.59; 95% CI, 0.53–0.65), and calibration, except PEWS-RL and SIRS (**Appendices 13** and **14**, http://links.lww.com/PCC/C445). For scores with multiple levels (i.e., PAWS, PEWS, and PEWS-IRISH), the increase in proportion of admissions meeting the primary outcome across lower levels was modest, indicating redundancy.

**Figure 1.**
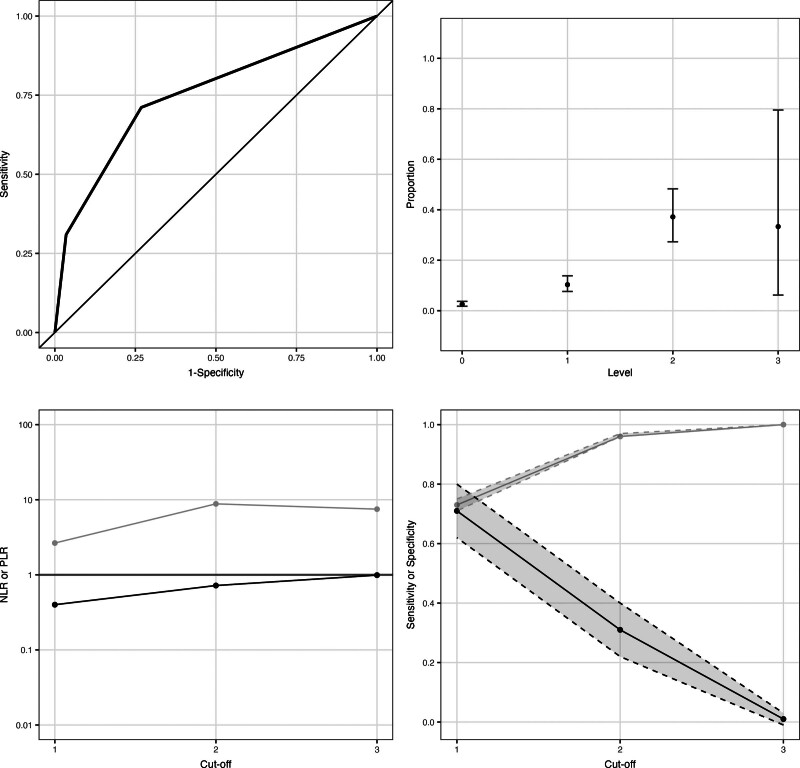
Summary performance measures of the quick Pediatric Logistic Organ Dysfunction-2 score. *Top left*: Discrimination (area under the receiver operating characteristic curve, 0.75; 95% CI, 0.70–0.80). *Top right*: Calibration. Proportion of admissions at each level of the score that died during their PICU stay; *error bars* indicate Wilson 95% CIs. *Bottom left*: Negative (*black line*) and positive (*gray line*) likelihood ratios at different cutoffs, illustrated on a log_10_ scale. *Bottom right*: Sensitivity (*black line*) and specificity (*gray line*) at different cutoffs; *gray shaded ribbons* indicate 95% CIs. NLR = negative likelihood ratio, PLR = positive likelihood ratio.

At a cutoff of greater than or equal to 1, the qPELOD-2 score demonstrated a sensitivity of 0.71 (95% CI, 0.62–0.80) and specificity of 0.73 (95% CI, 0.71–0.75). No other score achieved a sensitivity and specificity greater than 0.70 at any cutoff (**Appendix 15**, http://links.lww.com/PCC/C445). Three scores demonstrated modest potential for stratifying PICU admissions into low- and high-risk groups (**Appendix 16**, http://links.lww.com/PCC/C445): qPELOD-2 greater than or equal to 1 (PLR, 2.65; 95% CI, 2.28–3.09 and NLR, 0.40; 95% CI, 0.29–0.54), qSOFA greater than or equal to 2 (PLR, 2.97; 95% CI, 2.52–3.50 and NLR, 0.42; 95% CI, 0.31–0.56), and PAWS greater than or equal to 5 (PLR, 2.40; 95% CI, 2.04–2.82 and NLR, 0.46; 95% CI, 0.34–0.61). Summary measures of the best-performing score (qPELOD-2) are shown in **Figure 1**.

### Derivation of a New Prediction Model for Resource-Constrained PICU Contexts

Assessment of the relationship between continuous predictors and the primary outcome did not identify serious violations of linearity (**Appendix 17**, http://links.lww.com/PCC/C445). Age-dependent relationships between the primary outcome and heart rate and respiratory rate were evident (LRT; *p* < 0.001). There was no evidence of interaction between Spo_2_ and use of supplemental oxygen (LRT; *p* = 0.92). The full model, including the formulae to calculate the probability that a PICU admission will result in death, is shown in **Table 1**.

**TABLE 1.** Prognostic Model to Estimate the Probability That a PICU Admission Will End in Death

Clinical Domain	Predictors	Ridge Regression Coefficient^g^	*p*
	Intercept	–1.8525	
Background	Age (mo)	–0.0013	0.02
	Presence of comorbidity^a^	0.2201	0.12
	Weight-for-age *z* score^b^	–0.1108	< 0.001
Illness journey	Estimated travel time (min)^c^	0.0013	0.01
	Intra-hospital transfer^d^	0.4626	< 0.001
Cardiovascular	Heart rate (beats/min)	–0.0002	0.89
	Heart rate (beats/min) × age (mo)	8.6514 x10^–6^	0.14
	Prolonged capillary refill time^e^	1.0244	< 0.001
Respiratory	Respiratory rate (beats/min)	0.0090	0.01
	Respiratory rate (beats/min) × age (mo)	4.8939 x10^–5^	0.03
	Oxygen saturation (%)	–0.0253	< 0.001
	Receipt of supplemental oxygen	0.1808	0.10
Neurologic	Abnormal mental state^f^	1.0313	< 0.001

The model estimates the log odds of death during a PICU admission, using the sum of the intercept and the predictors multiplied by their coefficients, according to the following equation:

$-1.8525-0.0013\times age+\left\{ \begin{array}{l} 0ifnocomorbidity\\ 0.2201ifcomorbidity \end{array}-\right.0.1108\times waz+0.0013\times traveltime+$

$\left\{ \begin{array}{l} 0ifnointra\text{-}hopitaltransfer\\ 0.4626ifintra\text{-}hospitaltransfer \end{array}-\right.0.0002\times heartrate$ $+8.6514\times 10^{6}\times age\times heartrate+\left\{ \begin{array}{l} 0ifnoprolongedCRT\\ 1.0244ifprolongedCRT \end{array}\right.+$

$0.0090\times respiratoryrate+4.8939\times 10^{5}\times age\times respiratoryrate-0.0253\times oxygensaturation+$

$\left\{ \begin{array}{l} 0ifnosupplementaloxygen\\ 0.1808ifsupplementaloxygen \end{array}+\left\{ \begin{array}{l} 0ifnormalmentalstatus\\ 1.0313ifabnormalmentalstatus \end{array}\right.\right.$

To support clinical decision-making, the output of the model (log odds) is converted into the probability that a PICU admission will result in death using the following transformation:

$Pr\left(deathduringPICUadmission\right)=\frac{e^{logodds}}{1+e^{logodds}}$

a Assessed by a Principal Investigator (C.T.) blinded to outcome status using the following working definition: any previous health condition known to be present at PICU admission severe enough to require specialty pediatric care and probably a period of hospitalization over 12 mo (51).

b Calculated (R package: *zscorer*) (52) using World Health Organization (children < 10 yr) (53, 54) and U.S. Centers for Disease Control and Prevention (children ≥ 10 yr) (55) reference ranges. If weight was not recorded at PICU admission the closest value during the same hospital stay was used.

c Travel by road estimated using GoogleMaps.

d Admission from acute medical or surgical ward.

e Capillary refill time > 2 s.

f Glasgow Coma Scale < 15 and/or Alert/Voice/Pain/Unresponsiveness scale < A.

g Estimated using penalized (ridge) logistic regression; penalty parameter = 0.026 (R package: *ridge*) (32).

Predictors spanning the five clinical domains are presented along with their regression coefficients.

**Figure 2.**
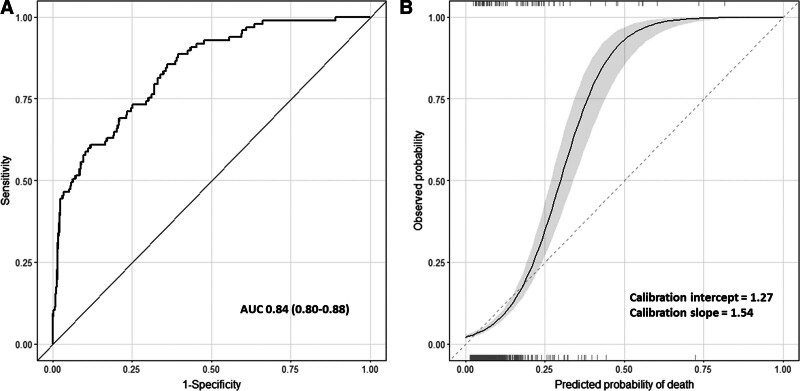
Discrimination and calibration of the new model. **A**, Discrimination of the new model. Perfect discrimination is indicated by an area under the receiver operating characteristic curve (AUC) of 1.0. **B**, Calibration of the new model. *Dashed line* indicates perfect calibration. *Solid line* indicates calibration of the model, with 95% CI (*gray ribbon*). *Rug plots* indicate distribution of predicted risks for participants who did (*top*) and did not (*bottom*) meet the primary outcome.

Discrimination of the new model (**Fig. 2*A***; AUC, 0.84; 95% CI, 0.80–0.88) was significantly better than all existing scores (DeLong test; *p* < 0.001). This is in part expected for a comparison between a newly derived model and external validation of existing scores. Calibration appeared best at lower predicted probabilities (**Fig. 2*B***), with the model underestimating risk for admissions with probabilities of death greater than 25%. Precision-recall curves for the new model and existing scores are shown in **Appendix 18** (http://links.lww.com/PCC/C445).

The ability of the model to triage PICU admissions into high- and low-acuity groups at cutoffs of 2.5%, 5%, 7.5%, 10%, and 15% is shown in **Table 2**; and **Appendix 19** (http://links.lww.com/PCC/C445). A cutoff of 10% reflects a triage strategy whereby all PICU admissions with a predicted probability of death greater than or equal to 10% are directed to a high-acuity area and all other PICU admissions are managed on the main unit. At this cutoff, PICU admissions triaged to the high-acuity area would have a probability of death almost five times that of the general PICU population (PLR, 5.75; 95% CI, 4.57–7.23), whereas the probability among those triaged to the low-acuity area would be less than half that of the general PICU population (NLR, 0.47; 95% CI, 0.37–0.59), and almost a tenth of those triaged to the high-acuity area. At the 10% cutoff, approximately 13.0% of all PICU admissions would be triaged to the high-acuity area, resulting in a ratio of 3:1 incorrect to correct (FP:TP) high-acuity triages.

**TABLE 2.** Ability of the Model to Triage PICU Admissions

Predicted Probability of Death	Positive Likelihood Ratio (95% CI)	Negative Likelihood Ratio (95% CI)	Per 1000 Admissions (~63 of Which Would Die)				Percentage of Admissions Triaged As High-Acuity (Ratio of Incorrect to Correct High-Acuity Triages)
			True Positive (High-Risk Admission Triaged to High-Acuity Area)	False Positive (Low-Risk Admission Triaged to High-Acuity Area)	True Negative (Low-Risk Admission Triaged to Low-Acuity Area)	False Negative (High-Risk Admission Triaged to Low-Acuity Area)	
2.5%	1.18 (1.14–1.21)	0.07 (0.01–0.46)	62	788	150	1	85.0% (13:1)
5%	2.31 (2.08–2.57)	0.23 (0.14–0.37)	54	347	590	9	40.1% (6:1)
7.5%	3.27 (2.73–3.91)	0.44 (0.33–0.57)	41	186	751	22	22.7% (5:1)
10%	5.75 (4.57–7.23)	0.47 (0.37–0.59)	36	94	844	26	13.0% (3:1)
15%	11.43 (8.22–15.88)	0.56 (0.46–0.67)	29	39	899	34	12.3% (1:1)

Performance of the model at five cutoffs (decision thresholds or threshold probabilities). A cutoff of 10% reflects a triage strategy whereby all admissions with a predicted probability of death ≥ 10% are directed to a high-acuity area and all other admissions managed on the main unit. A decrease in threshold probability (cutoff) is associated with an increase in the sensitivity of the triage strategy for identifying high-risk admissions, at the cost of a greater proportion of admissions being directed to the high-acuity area. Additional classification indices are provided in Appendix 18 (http://links.lww.com/PCC/C445).

### Generalizability and Applicability

**Figure 3.**
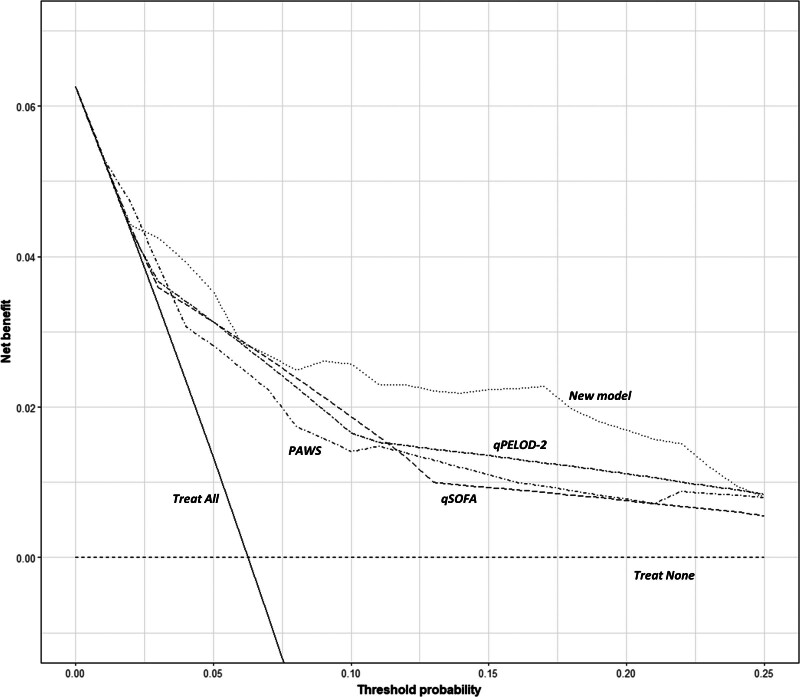
Clinical utility of the new model across a range of plausible decision thresholds. A cutoff (decision threshold or threshold probability) of 10% reflects a triage strategy whereby all admissions with a predicted probability of death greater than or equal to 10% are directed to a high-acuity area and all other admissions managed on the main unit. The net benefit of the new model (*dotted line*) is compared with “Treat All” (*solid line*; all PICU admissions are triaged to the high-acuity area) and “Treat None” (*shortdash*; no PICU admissions are triaged to the high-acuity area) strategies, as well as the three existing scores that demonstrated potential for stratifying admissions into low- and high-risk groups from the external validation (Pediatric Advanced Warning Score [PAWS] = *dot-shortdash*; quick Sequential Organ Failure Assessment [qSOFA] = *longdash*; quick Pediatric Logistic Organ Dysfunction-2 [qPELOD-2] = *dot-longdash*). Above a cutoff of 7.5% using the new model to triage admissions appears to be the optimal strategy. A color version of the figure is provided (Appendix 20, http://links.lww.com/PCC/C445) for additional clarity.

There is great heterogeneity in critical care provision across different resource-constrained contexts, with the relative value of a TP and FP depending on available resources. Decision curve analyses accounting for differing contexts indicate that using the model to support triage decisions could provide utility at cutoffs greater than or equal to 7.5% (**Fig. 3**; and **Appendix 20**, http://links.lww.com/PCC/C445), or simply put, in contexts where it might be desirable and feasible to manage up to a quarter (22.7%) of critical care admissions in a high-acuity area and tolerate up to 5:1 incorrect to correct (FP:TP) high-acuity triages.

## DISCUSSION

In this study, we report the ability of nine pediatric severity scores to risk stratify children at admission to a PICU in Cambodia and compared their performance to that of a novel prognostic model derived specifically for locations where critical care resources are scarce. Three scores (i.e., qPELOD-2, qSOFA, PAWS) had moderate diagnostic utility, but the new model proved superior and, if validated, could be used to support risk stratification of critically ill children in a variety of resource-constrained contexts.

This study provides one of few descriptions of pediatric critical care delivery in regions of LMICs with emerging critical care capacity. Children often had protracted illness journeys, consisting of multiple care encounters. The short average length of stay is striking and likely reflects the fact that in many resource-limited settings effective intensive care consists of providing simple, life-saving interventions for critically ill children with readily reversible conditions (1, 3). The ~4% post-PICU discharge mortality rate is likely an underestimate due to considerable losses to follow-up (37).

Except SIRS, which is known to perform poorly for risk assessment of acutely unwell children (38), the other eight scores had comparable discrimination. However, discrimination is a poor indicator of clinical utility (39). Only three scores were associated with meaningful changes in pre-test probability that a PICU admission might result in death, such that a single cutoff could be used to triage children into high- or low-risk groups. While separate cutoffs could identify high- and low-risk PICU admissions, in settings where resources are scarce it is unclear how a middle or “indeterminate” group might be managed, and dividing PICU admissions into multiple risk categories may not be practical.

Discrimination of FEAST-PET, qSOFA, and qPELOD-2 were comparable to their original development studies (18, 23, 38), which may reflect similarities in the population (i.e., critically ill children), mortality outcome, and for the FEAST-PET study, contextual factors (i.e., access to care, etc.). Performance of LqSOFA was inferior to the original development study (19), which is not unexpected since LqSOFA was devised for screening outside the PICU (19, 40). We note that discrimination and classification of PAWS, PEWS, and PEWS-RL were considerably worse in our study (19–22). These are “diagnostic” scores developed to predict events within 24 hours of calculation, and it is therefore unsurprising that prognostication of outcomes days later is suboptimal.

The model developed in our study considers the child’s background, illness journey, and vital organ function to contextualize assessment of critical illness and estimate probability of death before PICU discharge, given the resources available in a level II PICU outside a major urban center in Southeast Asia. Discrimination of our model was better than all nine severity scores. It provided good diagnostic value and was well calibrated over the threshold probabilities (cutoffs) of interest. In contexts where it might be feasible to resource a particular clinical area to manage up to a quarter of the highest risk PICU admissions, the model, if validated, could identify children who might benefit most from being cared for in such an area. Importantly, as the output of the model is continuous (as opposed to discrete as is the case for point-based scores), the cutoff for triage to the high-acuity area could be tailored to account for staffing availability, seasonal bed-pressures, hospital policy, and other dynamic contextual factors. Of note, no risk prediction model should replace clinical assessment; rather it could help healthcare professionals efficiently organize care for critically ill children.

In our study, best-practice methods were followed in model development, with particular care taken to prespecify and limit the number of candidate predictors and use penalized regression to avoid overfitting (14). Our analytical approach acknowledged that the relative importance of a TP and FP is context-dependent. Important contextual determinants of outcome were included in the model and likely contributed to its promising performance.

The main limitation of the model is the lack of external validation, which would be needed before it can be recommended for clinical use. A prospective validation study is underway in which the out-of-sample performance of the model will be compared with the best-performing of the existing severity scores. Although the model appears to underestimate the risk of death at predicted probabilities greater than or equal to 25%, miscalibration in this range is likely to be inconsequential (i.e., unlikely to affect a child’s triage category), as it is implausible that decision thresholds would be set so high, even in the most resource-constrained context. The study was conducted in a single center and hence findings may not be applicable to all resource-limited PICUs, particularly those in which causes of critical illness differ. Due to the retrospective nature of this study, travel time was estimated based on a child’s location of residence. This may not reflect actual travel time. It was not possible to evaluate the performance of all longlisted scores. In particular, only two of the nine included scores were developed in LMICs and it is disappointing that seven LMIC-derived scores had to be excluded (18, 22). The use of routine records means that measurement of clinical parameters was not standardized. However, use of clinical records did ensure the new model contains predictors feasible for collection and will hopefully increase the likelihood of successful out-of-sample validation.

## CONCLUSIONS

This study presents a new prognostic model for estimating the probability that a child admitted to a PICU in a resource-constrained context will not survive to discharge. The model contains predictors from multiple domains to ensure holistic assessment of critical illness. It outperformed nine existing severity scores and, if validated, offers a readily implementable and flexible mechanism to support risk stratification of critically ill children in resource-constrained contexts.

## ACKNOWLEDGMENTS

We are grateful to Soputhy Chansovannara and Phann Ysoun for their assistance with data collection, to Real Sophanith for her assistance with data entry, and to Thatsanun Ngernseng for setting up the study database.

## Supplementary Material


