# Supplementary material for: A Prognostic Model for Critically Ill Children in Locations With Emerging Critical Care Capacity*
Source: Pediatr Crit Care Med. 2023 Nov 10;25(3):189–200. doi: 10.1097/PCC.0000000000003394 (PMC10904005; doi:10.1097/PCC.0000000000003394)
Supplement: Supplementary file 1 [file pcc-25-0189-s001.docx]

**A prognostic model for critically ill children in locations with emerging critical care capacity**

**SUPPLEMENTARY APPENDIX**

**CONTENTS PAGE**

1. **TRIPOD checklist 2**
2. **Clinical proforma in routine use at the study site 3**
3. **Severity scores excluded at longlisting 8**
4. **Severity scores selected for external validation 10**
5. **Sensitivity analyses for primary outcome 11**
6. **Missing data patterns and results of sensitivity analyses 12**
7. **Study flowchart 14**
8. **Baseline characteristics 15**
9. **Maps depicting location of residence of children admitted to PICU 19**
10. **Supplementary clinical information about the cohort 20**
11. **UpSet plot illustrating clinical diagnoses amongst participants that died 21**
12. **Survival curves indicating time to meeting the primary and secondary outcomes 22**
13. **Discrimination of existing severity scores 23**
14. **Calibration of existing severity scores 24**
15. **Sensitivity and specificity of existing severity scores 25**
16. **Negative and positive likelihood ratios of existing severity scores 26**
17. **Relationship between continuous predictors and primary outcome 27**
18. **Precision-recall curves of the existing severity scores and new prediction model 28**
19. **Additional classification indices illustrating ability of the model to triage PICU admissions 29**
20. **Clinical utility of the new model (color version) 30**
21. **References 31**

**Appendix 1. TRIPOD checklist.**

| **Section/Topic** | **Item** |  | **Checklist Item** | **Page** |
| --- | --- | --- | --- | --- |
| **Title and abstract** | | | | |
| Title | 1 | D;V | Identify the study as developing and/or validating a multivariable prediction model, the target population, and the outcome to be predicted. | 1 |
| Abstract | 2 | D;V | Provide a summary of objectives, study design, setting, participants, sample size, predictors, outcome, statistical analysis, results, and conclusions. | 3 |
| **Introduction** | | | | |
| Background and objectives | 3a | D;V | Explain the medical context (including whether diagnostic or prognostic) and rationale for developing or validating the multivariable prediction model, including references to existing models. | 5 |
|  | 3b | D;V | Specify the objectives, including whether the study describes the development or validation of the model or both. | 5 |
| **Methods** | | | | |
| Source of data | 4a | D;V | Describe the study design or source of data (e.g., randomized trial, cohort, or registry data), separately for the development and validation data sets, if applicable. | 6 |
|  | 4b | D;V | Specify the key study dates, including start of accrual; end of accrual; and, if applicable, end of follow-up. | 6 |
| Participants | 5a | D;V | Specify key elements of the study setting (e.g., primary care, secondary care, general population) including number and location of centers. | 6 |
|  | 5b | D;V | Describe eligibility criteria for participants. | 6 |
|  | 5c | D;V | Give details of treatments received, if relevant. | 6 |
| Outcome | 6a | D;V | Clearly define the outcome that is predicted by the prediction model, including how and when assessed. | 7 |
|  | 6b | D;V | Report any actions to blind assessment of the outcome to be predicted. | 6 |
| Predictors | 7a | D;V | Clearly define all predictors used in developing or validating the multivariable prediction model, including how and when they were measured. | 6-7 |
|  | 7b | D;V | Report any actions to blind assessment of predictors for the outcome and other predictors. | 6 |
| Sample size | 8 | D;V | Explain how the study size was arrived at. | 7-8 |
| Missing data | 9 | D;V | Describe how missing data were handled (e.g., complete-case analysis, single imputation, multiple imputation) with details of any imputation method. | 7 |
| Statistical analysis methods | 10a | D | Describe how predictors were handled in the analyses. | 8 |
|  | 10b | D | Specify type of model, all model-building procedures (including any predictor selection), and method for internal validation. | 8 |
|  | 10c | V | For validation, describe how the predictions were calculated. | 8 |
|  | 10d | D;V | Specify all measures used to assess model performance and, if relevant, to compare multiple models. | 8-9 |
|  | 10e | V | Describe any model updating (e.g., recalibration) arising from the validation, if done. | N/A |
| Risk groups | 11 | D;V | Provide details on how risk groups were created, if done. | 8 |
| Development vs. validation | 12 | V | For validation, identify any differences from the development data in setting, eligibility criteria, outcome, and predictors. | Appendix 4 |
| **Results** | | | | |
| Participants | 13a | D;V | Describe the flow of participants through the study, including the number of participants with and without the outcome and, if applicable, a summary of the follow-up time. A diagram may be helpful. | 10 |
|  | 13b | D;V | Describe the characteristics of the participants (basic demographics, clinical features, available predictors), including the number of participants with missing data for predictors and outcome. | 10 |
|  | 13c | V | For validation, show a comparison with the development data of the distribution of important variables (demographics, predictors and outcome). | N/A |
| Model development | 14a | D | Specify the number of participants and outcome events in each analysis. | 10 |
|  | 14b | D | If done, report the unadjusted association between each candidate predictor and outcome. | N/A |
| Model specification | 15a | D | Present the full prediction model to allow predictions for individuals (i.e., all regression coefficients, and model intercept or baseline survival at a given time point). | Table 1 |
|  | 15b | D | Explain how to the use the prediction model. | Table 1 |
| Model performance | 16 | D;V | Report performance measures (with CIs) for the prediction model. | 11-12 |
| Model-updating | 17 | V | If done, report the results from any model updating (i.e., model specification, model performance). | N/A |
| **Discussion** | | | | |
| Limitations | 18 | D;V | Discuss any limitations of the study (such as nonrepresentative sample, few events per predictor, missing data). | 14 |
| Interpretation | 19a | V | For validation, discuss the results with reference to performance in the development data, and any other validation data. | 13 |
|  | 19b | D;V | Give an overall interpretation of the results, considering objectives, limitations, results from similar studies, and other relevant evidence. | 13-15 |
| Implications | 20 | D;V | Discuss the potential clinical use of the model and implications for future research. | 14-15 |
| **Other information** | | | | |
| Supplementary information | 21 | D;V | Provide information about the availability of supplementary resources, such as study protocol, Web calculator, and data sets. | 16 |
| Funding | 22 | D;V | Give the source of funding and the role of the funders for the present study. | 1 |

**Appendix 2. Clinical proforma in routine use at the study site.**

**
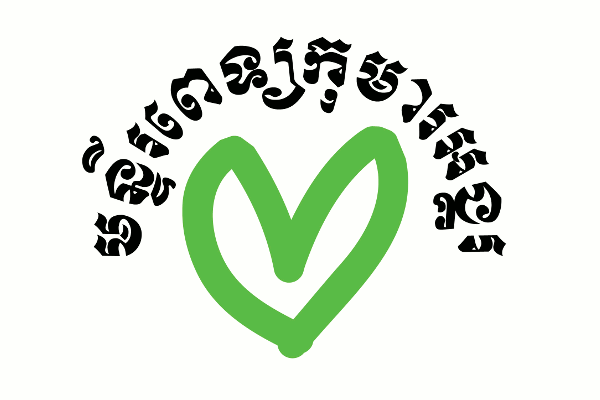
**

**Angkor Hospital for Children Admission Form**

*Patient Label with Address*

*ICU 2022*

**Date:** ____/____/____ **Time**____________

**Doctor: ______________________________________**

**Chief Complaint**.............................................................................................................................................

**History** …...……………………………………………………………………………………………………..................................................................... **.**.................................................................................................................................................................................................................................. …………………………………………..........................................................................................................................................................................

**Past Medical History / Birth History** …………………………………………………………………….................................................................. ................................................................................................................................................................................................................................... ...................................................................................................................................................................................................................................

**Current Medications**……………………………………………………………………….........................................................................

.............................................................................................................................................................................................................

**Past Medications**…………………………………………………………………………………………………..........................................

............................................................................................................................................................................................................

**Allergies** ………………………………………………………………………………………………………………………………………….

**Yellow Card Reviewed:** Yes, No **Growth Chart Plotted:**  Yes, No

**Immunizations:**

| **(Birth)** | **(6weeks)** | **(10weeks)** | **(14weeks)** | **(6months)** | **(9months)** | **(18months)** |  |
| --- | --- | --- | --- | --- | --- | --- | --- |
| BCG  HepB_0_ | DPT- HepB Hib_1_  PCV_1_  Polio_1_ | DPT- HepB Hib_2_  PCV_2_  Polio_2_ | DPT- HepB Hib_3_  PCV_3_  Polio_3_  IPV | MR_0_ | MR_1_  JE | MR_2_ | Unknown  Other:….  ……………  ……………. |

**Development History**

**Gross Motor** head control-rolls-sits-crawls-walks-runs-stairs-jumps

**Fine Motor** eyes fix-reaches-transfers-unilateral reach-pincer-throws-dresses

**Speech/Hearing** alert to sound –coos-laughs-babbles-1 word-2 words-4 to 6 words-many words-asked questions

**Social** looks at face-social smile-recognizes parent-explores-uses spoons & cup-plays-continent urine and stool

Based on above is development appropriate for age? Yes, No.

**Further Development History**..................................................................................................................................................................................

**Social History** ………………………………………………………………………………………………......................................................................

…………………………………………………………………………………………………………………...………......………………………………………………………………………………………………………….......................…………………………...………......……………………………………..

**Family History** - female

- male

**Examination**

Appearance ……………………………………………………………………………………………………………...

Temperature ……… ˚C HR.......... RR........... BP......./....... O_2_ Sat......... RA or ....... L/mn O_2_

Wt ……… kg Height ............ cm Wt/Ht Z-score: ...............SD

**HEENT**

Head Fontanelle.............................................................................Lymphadenopathy.....................

Eyes R………………………………….L……………………………..Fundus....................................

Ears R………………………….............L……………………………..

Nose

Throat

Other .........................................................................................................................

**Cardiovascular System**

Pulse (circle) strong fair weak cannot detect

Heart Sounds ……………………………………………………………….

Capillary Refill Time ……………………………………………………………….

Other (JVP, precordium) ......................................................................................

......................................................................................

**Respiratory System**

Auscultation …………

Grunting/flaring …………

Indrawing …………

Other (percussion, tracheal position) ………………………………………

**Gastro-Intestinal System**

Inspection …………

Palpation …………

Organs …………

Bowel Sounds …………

Masses …………

Other (genitalia, rectum, hernia, ascites) ..............………….............

**Nervous System**

Mental Status ........................ Kernig’s ……………………

Neck Stiffness ……………….. GCS ………………………. (Use Chart Below)

Cranial Nerves ..................................................................................................................

|  | 1 | 2 | 3 | 4 | 5 | 6 |
| --- | --- | --- | --- | --- | --- | --- |
| EYES | Does not open | Opens in response to pain | Opens in response to voice | Opens spontaneously | x | x |
| VERBAL | Makes no sounds | Incomprehensible | Says inappropriate words | Confused and disoriented | Oriented and converses normally | x |
| MOTOR | No movements | Extension to pain | Abnormal flexion to pain | Flexion/Withdrawal to pain | Localizes painful stimuli | Obeys commands |

Peripheral Nerves **R** arm **L** arm **R** leg **L** leg

Tone………………………………………………………………………............

Power……………………………………………………………………………….

Reflexes……………………………………………………………………………

Sensation…………………………………………………………………………..

Clonus ....................

Babinski ...................

Development .................................................................................................................................

…………………………………………………………………………………………………………………...


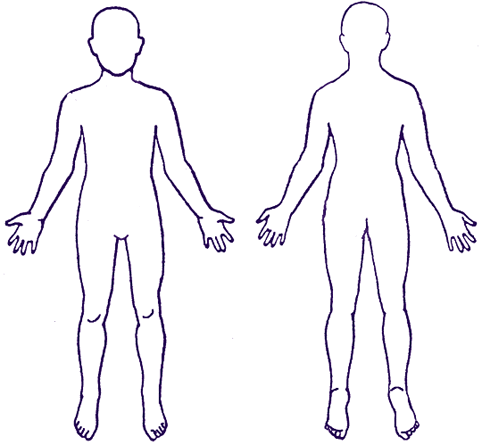


**Skin & Extremities**

Rash ………………………………..

Edema ………………………………..

Wounds ………………………………..

Other ………………………………..

………………………………..

**Other**…………………………………………………………………………………………………………………..….…………………………………………………………………………………………………………………………..…

………………………………………………………………………………………………………………………..…

**
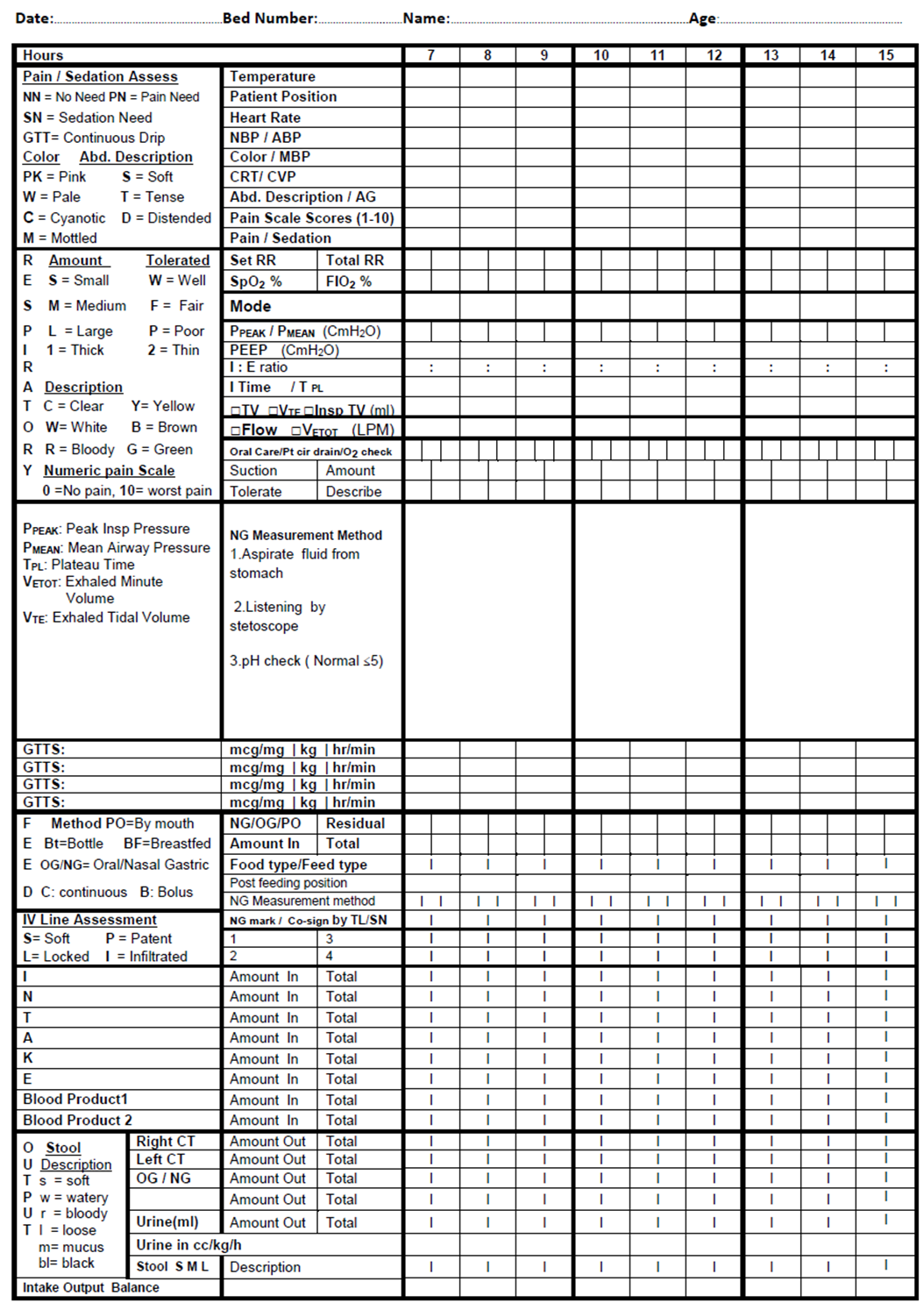
**

**Appendix 3. Severity scores** **excluded at longlisting.** Severity scores identified from two recent systematic reviews and PubMed search. Scores were excluded if they contained advanced diagnostic tests unlikely to be available in resource-constrained contexts (n = 15), included variables that were not relevant for the intended setting of use (n = 14), the information required to calculate the score/model was not provided in the original manuscript (n = 5), or the required variables were not available in the routine clinical records at the study site and no suitable proxy variable could be identified (n = 8). APTT = activated partial thromboplastin clotting time; ARI = acute respiratory infection; BUN = blood urea nitrogen; GE = gastroenteritis; HIV = human immunodeficiency virus; LDH = lactate dehydrogenase; IPSCC = international pediatric sepsis consensus conference; MUAC = mid-upper arm circumference; paCO_2_ = partial pressure of carbon dioxide in arterial blood; paO_2_ = partial pressure of oxygen in arterial blood; PCT = procalcitonin; PICU = pediatric intensive care unit; PT = prothrombin time; RA = room air; SpO_2_ = oxygen saturation.

| **NAME OF SCORE** | **ADVANCED DIAGNOSTIC TEST REQUIRED** | **INAPPROPRIATE FOR SETTING AND/OR POPULATION** | **DATA NOT AVAILABLE** | **REASONS FOR EXCLUSION** |
| --- | --- | --- | --- | --- |
| **AQUAMAT** | Y | N | N | BUN and base deficit required |
| **BITWE MODEL** | Y | N | Y | MUAC, infectious diagnosis (ARI, GE, malaria, bacteremia, other) required |
| **BITWE SCORE** | Y | N | Y | MUAC, infectious diagnosis (ARI, GE, malaria, bacteremia, other) required |
| **DRAMAIX** | Y | N | Y | Albumin, transthyretin, oedema, and MUAC required |
| **ELSHOUT** | N | Y | N | Sore throat, palpable lymphadenopathy not suitable for PICU population |
| **ERDMAN** | Y | N | N | Host biomarker tests required |
| **FEAST-PETaL** | Y | N | N | BUN, pH, and lactate required |
| **ITAT** | N | Y | N | SpO_2_ on RA not relevant for PICU population |
| **KWIZERA 1** | N | N | N | Information not available for construction of the score/model |
| **KWIZERA 2** | N | N | N | Information not available for construction of the score/model |
| **KWIZERA 3** | N | N | N | Information not available for construction of the score/model |
| **KWIZERA 4** | N | N | N | Information not available for construction of the score/model |
| **KWIZERA 5** | N | N | N | Information not available for construction of the score/model |
| **LIN NOMOGRAM** | Y | N | N | Blood culture, albumin, and LDH required |
| **LODS** | N | N | Y | Deep breathing and prostration required |
| **LOWLAAVAR 1** | N | Y | N | HIV not relevant (low endemicity) |
| **LOWLAAVAR 2** | N | Y | Y | HIV not relevant (low endemicity), MUAC required |
| **LOWLAAVAR 3** | N | N | Y | MUAC required - and as this is a model cannot substitute a proxy variable |
| **MPIMBAZA** | N | N | Y | Prostration, jaundice, deep breathing, and meningitic signs required |
| **mPRIO** | Y | Y | Y | SpO_2_ on RA, PCT, organ dysfunction as per IPSCC definition |
| **mRISC** | N | Y | Y | Malaria not relevant (low endemicity), dehydration, prostration, night sweats, and historical loss of consciousness required |
| **PCIS** | Y | N | Y | BUN, creatinine, K^+^, Na^+^, pH, paO_2_, and gastrointestinal bleeding required |
| **PEDIA-e** | N | N | Y | Prostration, jaundice, and kwashiorkor required |
| **PEDIA-i** | N | N | Y | Deep breathing, prostration, and jaundice required |
| **PEDIA-l** | N | N | Y | Prostration and kwashiorkor required |
| **PELOD-2** | Y | N | Y | Lactate, creatinine, paO_2_, paCO_2_, and pupillary reaction required |
| **PERCH** | N | Y | Y | SpO_2_ on RA not relevant, deep breathing, cough, and grunting required |
| **PEWS BCH** | N | N | Y | Skin color, frequency of nebulization prior to assessment, nurse concern, and family concern required |
| **PIM III** | Y | Y | Y | Base excess, PaO2, and pupillary reactions required, along with many other high-income country contextual variables |
| **PIRO** | Y | Y | Y | paO_2_, BUN, transaminases, PT, blood culture, SpO_2_ on RA not relevant for PICU population, signs of liver failure |
| **pMODS** | Y | N | N | Lactate, bilirubin, paO_2_, fibrinogen, and BUN required |
| **PRISM III** | Y | N | Y | pCO_2_, paO_2_, pH, acidosis, total CO_2_, K^+^, BUN, creatinine, PT/APTT, and pupillary reflexes required |
| **pSOFA** | Y | N | N | Bilirubin and creatinine required |
| **qSOFA-L** | Y | N | N | Lactate required |
| **RISC** | N | Y | Y | SpO_2_ on RA not relevant for PICU population and HIV not relevant (low endemicity), prostration, and wheezing required |
| **RISC-Malawi** | N | Y | Y | SpO_2_ on RA not relevant for PICU population, MUAC, and wheezing required |
| **SCOTT** | N | Y | Y | Arrival via emergency medical services, indwelling central line, and hospitalized within last year required |
| **SICK** | N | Y | N | SpO_2_ on RA not relevant for PICU population |
| **TORPS** | N | Y | N | SpO_2_ on RA not relevant for PICU population |
| **YOS** | N | N | Y | Quality of cry, reaction to parent stimulation, state variation, color, and response to social overtures required |

**Appendix 4. Severity scores selected for external validation.** Scores were selected for external validation irrespective of the setting, population, and outcome used for the original derivation study. The only prerequisites were that the score had to be calculable with the available data (with the exception that systolic blood pressure could be dropped if CRT was included),^1^ relevant to the study population, and feasible for implementation in a resource-limited PICU context. AVPU = Alert Voice Pain Unresponsive scale; CRT = capillary refill time; ED = emergency department; FEAST-PET = Fluid Expansion as Supportive Therapy-Pediatric Emergency Triage; GCS = Glasgow Coma Scale; LqSOFA = Liverpool quick Sequential Organ Failure Assessment; PAWS = Paediatric Advanced Warning Score; PEWS = Pediatric Early Warning System; PEWS-RL = PEWS-Resource Limited; PICU = pediatric intensive care unit; qPELOD-2 = quick Pediatric Logistic Organ Dysfunction-2; SIRS = systemic inflammatory response syndrome; SpO_2_ = oxygen saturation.

| **Score** | **Range** | **Predictors** | **Adjustments** | **Original setting, population, and outcome** |
| --- | --- | --- | --- | --- |
| **FEAST-PET** | 0-10 | Heart rate, temperature, CRT, pulse character, work of breathing, lung crepitations, mental status, pallor | Cut-off for CRT increased to ≥ 3 seconds; deep breathing omitted from work of breathing; age-adjusted WHO criteria for severe anemia used as a proxy for pallor;^2^ mental status dichotomized and assessed using AVPU or GCS, reducing the maximum possible score to 9 | Score to predict 48-hour mortality on admission to secondary and tertiary-care hospitals in East Africa in children with severe febrile illness^3^ |
| **LqSOFA** | 0-4 | Respiratory rate, heart rate, CRT, mental status | Mental status assessed using AVPU or GCS | Score to predict PICU admission or death in febrile children presenting to ED in the United Kingdom^1^ |
| **PAWS** | 0-21 | Respiratory rate, heart rate, temperature, CRT, mental status, SpO_2_, work of breathing | Mental status assessed using AVPU or GCS; CRT, mental status, and work of breathing dichotomized, reducing the maximum possible score to 17 | Score to predict need for PICU admission in children presenting to ED in the United Kingdom^4^ |
| **PEWS** | 0-26 | Respiratory rate, heart rate, systolic blood pressure, CRT, SpO_2_, supplemental oxygen, work of breathing | Systolic blood pressure omitted; work of breathing dichotomized, reducing the maximum possible score to 20 | Score to predict need for PICU admission in children hospitalized on a general pediatric ward in a tertiary-care hospital in Canada^5^ |
| **PEWS-IRISH** | 0-21 | Respiratory rate, heart rate, systolic blood pressure, CRT, mental status, SpO_2_, supplemental oxygen, work of breathing | Systolic blood pressure omitted; work of breathing and mental status dichotomized, reducing the maximum possible score to 15 | Adaptation of the PEWS score by the Irish National Clinical Effectiveness Committee^6^ |
| **PEWS-RL** | 0-6 | Respiratory rate, heart rate, temperature, mental status, supplemental oxygen, work of breathing |  | Score to predict clinical deterioration in children hospitalized on a general pediatric ward in a tertiary-care hospital in Rwanda^7^ |
| **qPELOD-2** | 0-3 | Heart rate, systolic blood pressure, mental status | CRT used as a proxy for systolic blood pressure; mental status assessed using AVPU or GCS | Score to predict mortality in children with suspected infection on admission to nine European PICUs^8^ |
| **qSOFA** | 0-3 | Respiratory rate, systolic blood pressure, mental status | CRT used as a proxy for systolic blood pressure; mental status assessed using AVPU or GCS | Adult sepsis score to predict mortality adapted for children with suspected infection on admission to PICUs in Australia and New Zealand^9^ |
| **SIRS** | 0-4 | Respiratory rate, heart rate, temperature, white cell count |  | Expert consensus definition for the diagnosis of pediatric sepsis^10^ |

**Appendix 5. Sensitivity analyses for primary outcome.** Top table: comparison between the primary analysis (n = 1,550; outcome events = 97) and a sensitivity analysis (n = 1529; outcome events = 76) where admissions which met the primary outcome but in which the death was judged to have been related to a second illness acquired during the PICU stay (n = 9) and admissions which were discharged to die at home (n = 12) were excluded. Bottom table: comparison between the primary analysis and a sensitivity analysis in which laboratory parameters were restricted to those available between two hours prior and four hours after PICU admission.^11^ Results are presented for the two scores which included laboratory parameters: FEAST-PET (hemoglobin as a proxy for pallor) and SIRS (white cell count). In the sensitivity analysis, with the more restrictive criteria for inclusion of laboratory parameters, missingness (addressed by median imputation grouped by outcome status as for the primary analysis) increased to 30.2% for FEAST-PET and 31.0% for SIRS.

| **MODEL / SCORE** | **AUC (95% CI)** | |
| --- | --- | --- |
|  | **PRIMARY ANALYSIS** | **SENSITIVITY ANALYSIS** |
| **NEW MODEL** | 0.84 (0.80-0.88) | 0.83 (0.78-0.88) |
| **FEAST-PET** | 0.72 (0.66-0.78) | 0.74 (0.67-0.80) |
| **LqSOFA** | 0.76 (0.71-0.81) | 0.79 (0.74-0.84) |
| **PAWS** | 0.76 (0.71-0.81) | 0.77 (0.72-0.82) |
| **PEWS** | 0.71 (0.65-0.76) | 0.72 (0.66-0.79) |
| **PEWS-IRISH** | 0.74 (0.69-0.79) | 0.74 (0.69-0.80) |
| **PEWS-RL** | 0.72 (0.67-0.77) | 0.72 (0.67-0.78) |
| **qPELOD-2** | 0.75 (0.70-0.80) | 0.76 (0.70-0.81) |
| **qSOFA** | 0.74 (0.69-0.79) | 0.74 (0.69-0.80) |
| **SIRS** | 0.59 (0.53-0.65) | 0.62 (0.55-0.68) |

| **MODEL / SCORE** | **AUC (95% CI)** | |
| --- | --- | --- |
|  | **PRIMARY ANALYSIS** | **SENSITIVITY ANALYSIS** |
| **FEAST-PET** | 0.72 (0.66-0.78) | 0.72 (0.66-0.78) |
| **SIRS** | 0.59 (0.53-0.65) | 0.58 (0.52-0.63) |

**Appendix 6. Missing data patterns and results of sensitivity analyses.** Top figure: missingness pattern for existing severity scores evaluated in external validation. Bottom figure: missingness pattern for candidate predictors included in new clinical prediction model. Table: results of sensitivity analyses conducted using different approaches for handling missing data. For best case imputation, missing values amongst admissions that met the primary outcome were assigned the most extreme values in the dataset, whilst missing values amongst admissions that did not meet the primary outcome were assigned a normal value (e.g. median heart rate, 100% oxygen saturation, no supplemental oxygen, etc.) The opposite approach was taken for worst case imputation, with missing values amongst admissions that met the primary outcome being assigned a normal value and missing values amongst admissions that did not meet the primary outcome being assigned an extreme value.


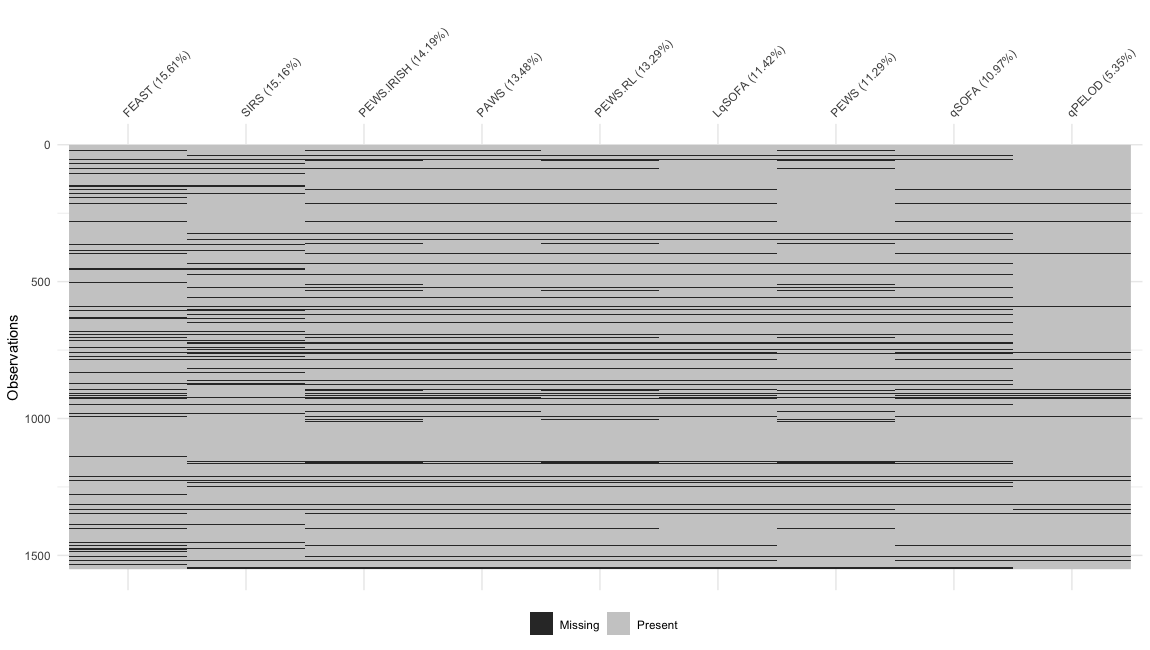


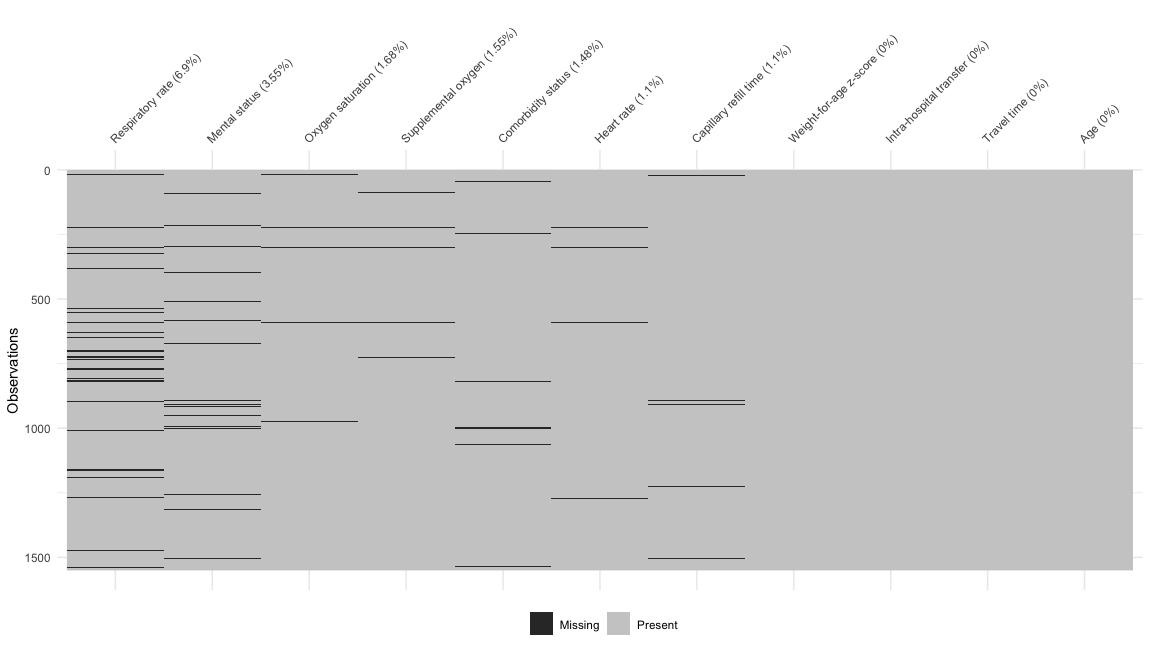


| **MODEL / SCORE** | **AUC (95% CI)** | | | |
| --- | --- | --- | --- | --- |
|  | **COMPLETE CASE** | **MEDIAN IMPUTATION** | **BEST CASE** | **WORST CASE** |
| **NEW MODEL** | 0.83 (0.79-0.88) | 0.84 (0.80-0.88) | 0.86 (0.83-0.90) | 0.78 (0.74-0.83) |
| **FEAST-PET** | 0.67 (0.60-0.74) | 0.72 (0.66-0.78) | 0.74 (0.68-0.80) | 0.67 (0.61-0.73) |
| **LqSOFA** | 0.75 (0.69-0.80) | 0.76 (0.71-0.81) | 0.78 (0.73-0.83) | 0.71 (0.66-0.76) |
| **PAWS** | 0.73 (0.67-0.78) | 0.76 (0.71-0.81) | 0.78 (0.73-0.83) | 0.70 (0.65-0.75) |
| **PEWS** | 0.67 (0.61-0.73) | 0.71 (0.65-0.76) | 0.73 (0.67-0.78) | 0.67 (0.62-0.73) |
| **PEWS-IRISH** | 0.69 (0.63-0.75) | 0.74 (0.69-0.79) | 0.76 (0.71-0.81) | 0.69 (0.64-0.74) |
| **PEWS-RL** | 0.68 (0.62-0.74) | 0.72 (0.67-0.77) | 0.73 (0.68-0.78) | 0.66 (0.61-0.71) |
| **qPELOD-2** | 0.73 (0.68-0.79) | 0.75 (0.70-0.80) | 0.75 (0.70-0.80) | 0.70 (0.64-0.75) |
| **qSOFA** | 0.72 (0.66-0.77) | 0.74 (0.69-0.79) | 0.74 (0.69-0.79) | 0.70 (0.64-0.75) |
| **SIRS** | 0.62 (0.55-0.68) | 0.59 (0.53-0.65) | 0.63 (0.57-0.68) | 0.56 (0.50-0.62) |

**Appendix 7. Study flowchart.**


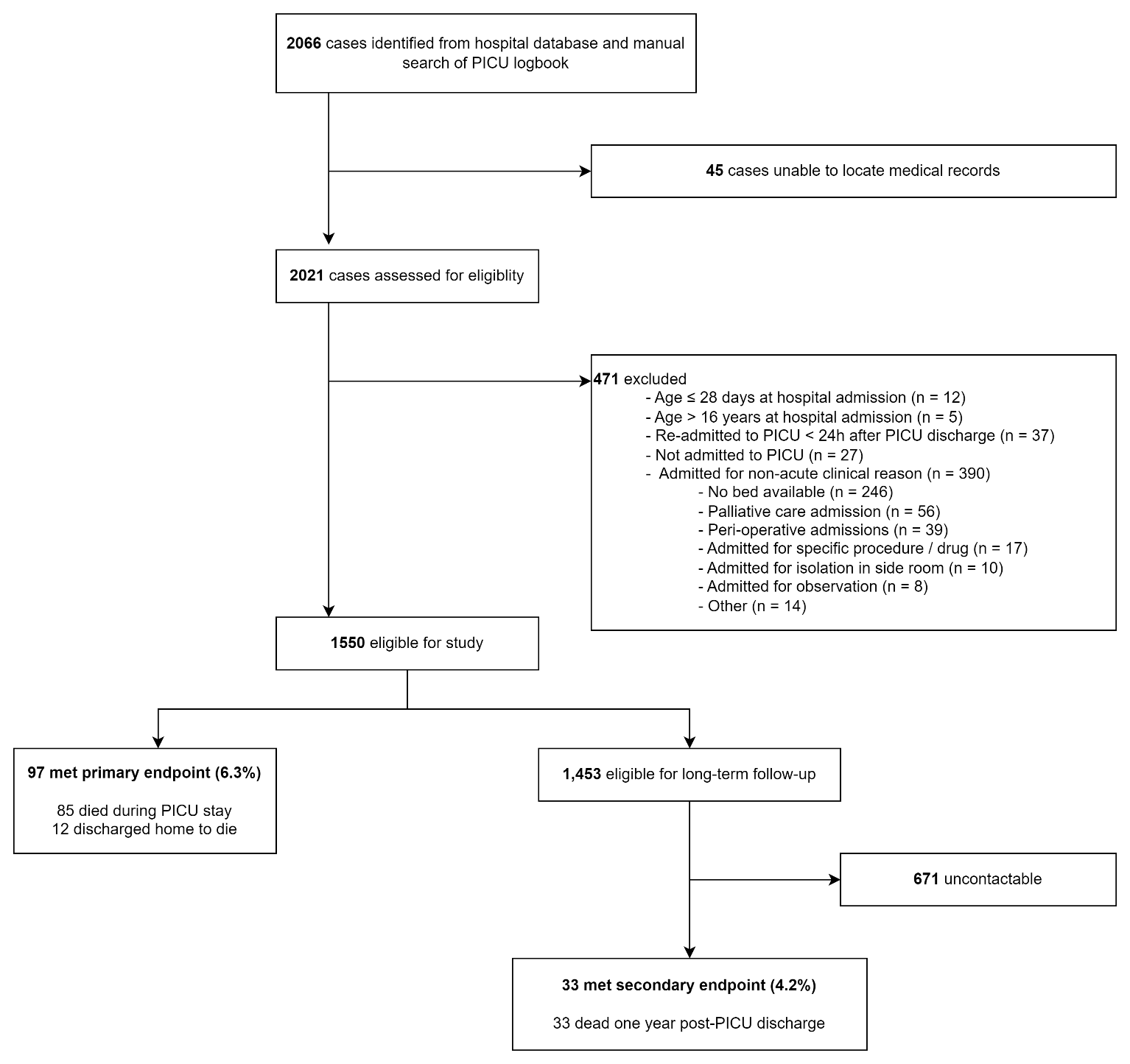


**Appendix 8. Baseline characteristics.** Baseline demographic, background, illness history, anthropometric, clinical, and laboratory characteristics of the cohort, stratified by primary outcome status. ^a^Missing data: comorbidity = 23; preterm birth = 84; low birthweight = 238; illness duration = 2; number of previous care encounters = 391; axillary temperature = 33; heart rate = 17; respiratory rate = 107; oxygen saturation = 26; supplemental oxygen = 24; mental status = 55; CRT = 17; pulse character = 42; cool extremities = 10; respiratory distress = 22; lung crackles = 10; white cell count = 117; neutrophil count = 117; lymphocyte count = 118; hemoglobin = 114; platelet count = 115; C-reactive protein = 411; glucose = 386; LqSOFA = 177; qSOFA = 170; qPELOD-2 = 83; SIRS = 235; PEWS = 175; PEWS-RL = 206; PEWS-IRISH = 220; PAWS = 209; FEAST-PET = 242. ^b^Baseline SpO_2_ amongst those not receiving supplemental oxygen at the time of PICU admission confirmed a similar relationship (96.5% vs. 98.0%; p < 0.001; n = 798). ^c^Not alert = GCS < 15 or AVPU < A; prolonged CRT defined as > 2 seconds; respiratory distress = chest indrawing, tracheal tug, or nasal flaring. ^d^Laboratory parameters included if measured within 24 hours of PICU admission. AVPU = Alert Voice Pain Unresponsiveness scale; Bpm = beats/breaths per minute; CRT = capillary refill time; GCS = Glasgow Coma Scale; PICU = pediatric intensive care unit.

| **Characteristic** | **Overall**, N = 1,550^1^ | **PICU survival** | | |
| --- | --- | --- | --- | --- |
|  |  | **No**, N = 97^1^ | **Yes**, N = 1,453^1^ | **p-value**^2^ |
| **Demographics** |  |  |  |  |
| **Age (months)** | 14.0 (4.0, 73.0) | 18.0 (6.0, 60.0) | 13.0 (4.0, 73.0) | 0.30 |
| **Male sex** | 927 / 1,550 (60%) | 54 / 97 (56%) | 873 / 1,453 (60%) | 0.40 |
| **Known comorbidity**^a^ | 266 / 1,527 (17%) | 27 / 94 (29%) | 239 / 1,433 (17%) | 0.003 |
| **Perinatal history** |  |  |  |  |
| **Reported preterm birth**^a^ | 111 / 1,466 (7.6%) | 7 / 87 (8.0%) | 104 / 1,379 (7.5%) | 0.90 |
| **Reported low birthweight**^a^ | 193 / 1,312 (15%) | 13 / 75 (17%) | 180 / 1,237 (15%) | 0.50 |
| **Location of residence** |  |  |  |  |
| **Travel time to hospital (minutes)** | 69.0 (27.0, 156.0) | 88.0 (50.0, 197.0) | 69.0 (27.0, 156.0) | 0.008 |
| **Distance to hospital (kilometers)** | 60.8 (14.8, 147.0) | 80.8 (31.4, 178.0) | 60.8 (14.8, 139.0) | 0.006 |
| **Illness history** | |  |  |  |
| **Duration of illness (days)**^a^ | 3.0 (2.0, 5.0) | 3.0 (2.0, 7.0) | 3.0 (2.0, 5.0) | 0.07 |
| **Care sought prior to admission at study site** | 1,160 / 1,550 (75%) | 78 / 97 (80%) | 1,082 / 1,453 (74%) | 0.20 |
| Traditional healer | 30 / 1,160 (2.6%) | 3 / 78 (3.8%) | 27 / 1,082 (2.5%) | 0.40 |
| Government primary health center | 296 / 1,160 (26%) | 17 / 78 (22%) | 279 / 1,082 (26%) | 0.40 |
| Private pharmacy | 142 / 1,160 (12%) | 11 / 78 (14%) | 131 / 1,082 (12%) | 0.60 |
| Government hospital | 114 / 1,160 (9.8%) | 13 / 78 (17%) | 101 / 1,082 (9.3%) | 0.04 |
| Non-governmental healthcare provider | 133 / 1,160 (11%) | 15 / 78 (19%) | 118 / 1,082 (11%) | 0.03 |
| Private hospital/clinic | 449 / 1,160 (39%) | 34 / 78 (44%) | 415 / 1,082 (38%) | 0.40 |
| Study site | 171 / 1,160 (15%) | 13 / 78 (17%) | 158 / 1,082 (15%) | 0.60 |
| Satellite clinic of study site | 195 / 1,160 (17%) | 7 / 78 (9.0%) | 188 / 1,082 (17%) | 0.06 |
| Other healthcare provider | 49 / 1,160 (4.2%) | 3 / 78 (3.8%) | 46 / 1,082 (4.3%) | >0.90 |
| **Number of previous care encounters**^a^ | 1.0 (1.0, 2.0) | 1.0 (1.0, 2.0) | 1.0 (1.0, 2.0) | 0.30 |
| **Overnight admission prior to presentation** | 268 / 1,550 (17%) | 29 / 97 (30%) | 239 / 1,453 (16%) | <0.001 |
| **Inter-hospital transfer to study site** | 318 / 1,550 (21%) | 26 / 97 (27%) | 292 / 1,453 (20%) | 0.11 |
| Satellite clinic of study site | 194 / 318 (61%) | 8 / 26 (31%) | 186 / 292 (64%) | < 0.001 |
| Other | 108 / 318 (34%) | 12 / 26 (46%) | 96 / 292 (33%) | 0.20 |
| Self-transfer | 16 / 318 (5.0%) | 6 / 26 (23%) | 10 / 292 (3.4%) | < 0.001 |
| **Intra-hospital admission to PICU** | 294 / 1,550 (19%) | 32 / 97 (33%) | 262 / 1,453 (18%) | <0.001 |
| **Anthropometrics** |  |  |  |  |
| **Weight-for-age z-score** | -1.40 (-2.41, -0.53) | -1.81 (-3.37, -0.97) | -1.39 (-2.37, -0.51) | 0.003 |
| **Vital signs** |  |  |  |  |
| **Axillary temperature (°C)**^a^ | 36.9 (36.4, 37.6) | 37.0 (36.4, 37.7) | 36.9 (36.4, 37.6) | 0.90 |
| Fever (≥ 37.5°C) | 462 / 1,517 (30%) | 30 / 94 (32%) | 432 / 1,423 (30%) | 0.80 |
| Hypothermia (< 35.5°C) | 46 / 1,517 (3.0%) | 9 / 94 (9.6%) | 37 / 1,423 (2.6%) | 0.001 |
| **Heart rate (bpm)**^a^ |  |  |  |  |
| < 12 months | 165.0 (148.0, 176.0) | 160.0 (145.0, 176.8) | 165.0 (148.0, 176.0) | 0.50 |
| 12 to 59 months | 152.0 (132.0, 169.5) | 142.0 (124.0, 161.0) | 153.0 (134.0, 170.0) | 0.03 |
| 60 months to 12 years | 115.0 (98.0, 132.0) | 150.0 (132.0, 164.0) | 113.0 (98.0, 129.0) | <0.001 |
| > 12 years | 99.0 (83.0, 114.0) | 128.0 (100.8, 151.5) | 98.0 (81.0, 112.0) | 0.05 |
| **Respiratory rate (bpm)**^a^ |  |  |  |  |
| < 12 months | 56.0 (48.0, 62.0) | 56.0 (48.5, 68.0) | 56.0 (48.0, 62.0) | 0.60 |
| 12 to 59 months | 48.0 (38.0, 60.0) | 46.0 (38.0, 56.5) | 50.0 (38.0, 60.0) | 0.50 |
| 60 months to 12 years | 30.0 (26.0, 36.0) | 42.0 (32.0, 52.0) | 30.0 (26.0, 36.0) | <0.001 |
| > 12 years | 26.0 (24.0, 32.0) | 39.0 (35.8, 40.0) | 26.0 (24.0, 32.0) | 0.002 |
| **Oxygen saturation (%)**^a,b^ | 98.0 (96.0, 99.0) | 98.0 (89.2, 99.0) | 98.0 (97.0, 99.0) | <0.001 |
| **On supplemental oxygen**^a^ | 767 / 1,526 (50%) | 57 / 95 (60%) | 710 / 1,431 (50%) | 0.05 |
| **Clinical assessment**^c^ |  |  |  |  |
| **Not alert**^a^ | 351 / 1,495 (23%) | 56 / 89 (63%) | 295 / 1,406 (21%) | <0.001 |
| **Prolonged capillary refill time**^a^ | 126 / 1,533 (8.2%) | 31 / 97 (32%) | 95 / 1,436 (6.6%) | <0.001 |
| **Weak pulse**^a^ | 183 / 1,508 (12%) | 28 / 92 (30%) | 155 / 1,416 (11%) | <0.001 |
| **Cool extremities**^a^ | 406 / 1,540 (26%) | 43 / 97 (44%) | 363 / 1,443 (25%) | <0.001 |
| **Respiratory distress**^a^ | 900 / 1,528 (59%) | 67 / 92 (73%) | 833 / 1,436 (58%) | 0.01 |
| **Lung crepitations**^a^ | 561 / 1,540 (36%) | 34 / 95 (36%) | 527 / 1,445 (36%) | 0.90 |
| **Laboratory parameters**^d^ |  |  |  |  |
| **White cell count (x10^9^ cells/µL)**^a^ | 12.4 (8.2, 17.2) | 12.1 (6.2, 17.8) | 12.4 (8.4, 17.2) | 0.20 |
| Neutrophil count (x10^9^ cells/µL)^a^ | 6.0 (3.2, 10.1) | 5.8 (2.5, 9.5) | 6.0 (3.2, 10.1) | 0.40 |
| Lymphocyte count (x10^9^ cells/µL)^a^ | 3.7 (2.1, 6.2) | 3.1 (1.5, 6.0) | 3.7 (2.1, 6.3) | 0.05 |
| **Hemoglobin (g/dL)**^a^ | 108.0 (95.0, 122.0) | 96.0 (79.0, 108.0) | 109.0 (97.0, 123.0) | <0.001 |
| **Platelet count (x10^9^ cells/µL)**^a^ | 376.0 (214.5, 506.5) | 242.0 (76.0, 371.0) | 384.0 (229.2, 509.5) | <0.001 |
| **C-reactive protein (mg/L)**^a^ | 7.0 (2.0, 33.0) | 13.5 (2.0, 74.5) | 7.0 (2.0, 30.0) | 0.03 |
| **Glucose (mmol/L)**^a^ | 104.0 (86.0, 129.0) | 111.5 (86.5, 159.8) | 103.5 (86.0, 128.0) | 0.20 |
| **Severity scores** |  |  |  |  |
| **FEAST-PET**^a^ | 2.0 (1.0, 3.0) | 3.0 (2.0, 4.0) | 2.0 (1.0, 3.0) | <0.001 |
| **LqSOFA**^a^ | 0.0 (0.0, 1.0) | 1.0 (1.0, 2.0) | 0.0 (0.0, 1.0) | <0.001 |
| **PAWS**^a^ | 3.0 (2.0, 5.0) | 5.0 (3.0, 7.0) | 3.0 (2.0, 5.0) | <0.001 |
| **PEWS**^a^ | 6.0 (3.0, 8.0) | 7.5 (6.0, 11.0) | 5.0 (3.0, 8.0) | <0.001 |
| **PEWS-IRISH**^a^ | 4.0 (2.0, 7.0) | 6.0 (4.2, 8.8) | 4.0 (2.0, 6.0) | <0.001 |
| **PEWS-RL**^a^ | 3.0 (2.0, 3.0) | 3.0 (3.0, 4.0) | 3.0 (1.0, 3.0) | <0.001 |
| **qPELOD-2**^a^ | 0.0 (0.0, 1.0) | 1.0 (0.0, 2.0) | 0.0 (0.0, 1.0) | <0.001 |
| **qSOFA**^a^ | 1.0 (1.0, 1.0) | 2.0 (1.0, 2.0) | 1.0 (1.0, 1.0) | <0.001 |
| **SIRS**^a^ | 2.0 (1.0, 2.0) | 2.0 (1.0, 3.0) | 2.0 (1.0, 2.0) | <0.001 |
| ^1^Median (IQR); n / N (%); ^2^Wilcoxon rank sum test; Pearson's Chi-squared test; Fisher's exact test | | | | |

**
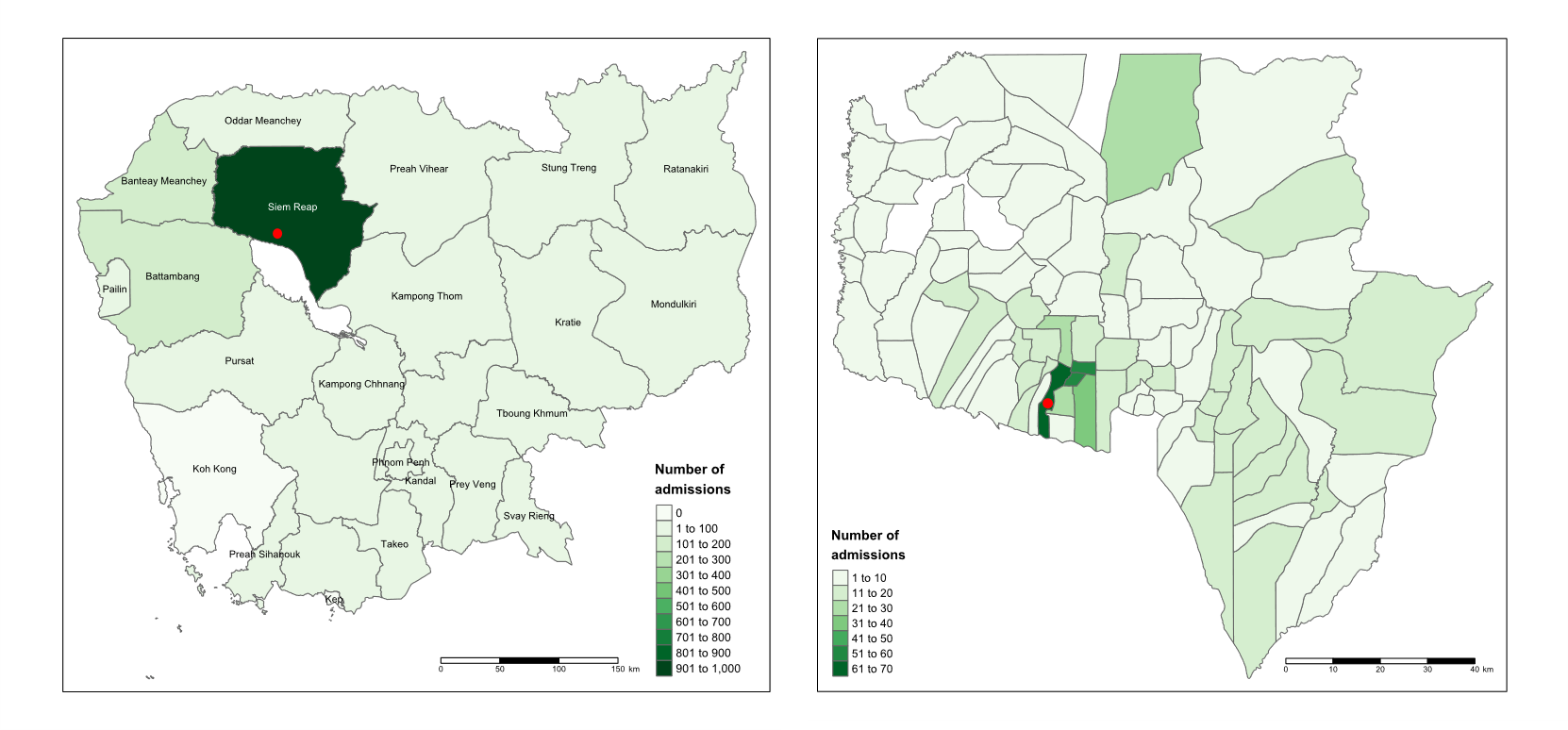
Appendix 9. Maps depicting locations of residence for children admitted to PICU.** Left panel: distribution of admissions across Cambodian provinces. Right panel: distribution of admissions across communes in Siem Reap province. The study site is indicated by the red dot.

**Appendix 10. Supplementary clinical information about the cohort.** Additional clinical information is provided to help readers understand the context of the study and consider potential generalizability to other settings with emerging critical care capacity. ^a^Defined as history of fever or temperature ≥ 37.5°C at PICU admission (missing data; n = 10).

| **Supplementary clinical information about the cohort** | |
| --- | --- |
| **Reasons for PICU admission**  Febrile illness^a^  Respiratory distress  Circulatory instability  Impaired consciousness | 79.1% (1,128/1,540)  63.8% (989/1,550)  35.2% (545/1,550)  22.7% (352/1,550) |
| **Vital organ support**  Any vital organ support  Non-invasive ventilation  Mechanical ventilation  Inotropic therapy  Peritoneal dialysis | 41.7% (647/1,550)  33.3% (516/1,550)  22.8% (354/1,550)  6.3% (98/1,550)  0.3% (4/1,550) |
| **Length of stay on PICU (days)** | 2 (1-4) |
| **Most frequent discharge diagnoses**  Pneumonia  Bronchiolitis  Dengue | 32.3% (501/1,550)  20.6% (319/1,550)  14.3% (221/1,550) |
| Median (IQR) provided for continuous variables; % (n / N) provided for categorical variables | |

**Appendix 11. UpSet plot illustrating clinical diagnoses amongst participants that died.** Sets contain children with a particular diagnosis. Intersections contain children with a particular combination of diagnoses.

**
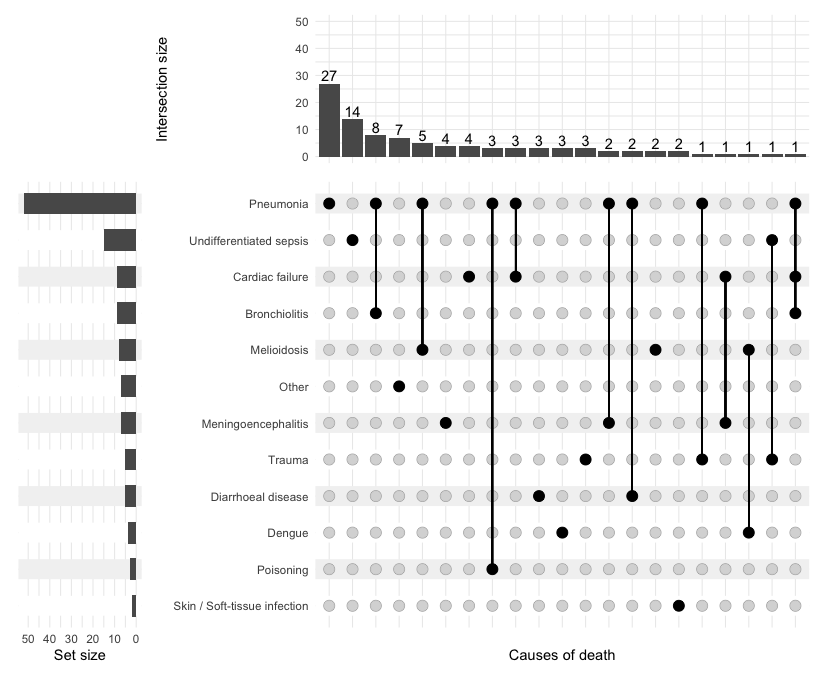
**

**Appendix 12. Time to meeting the primary and secondary outcomes.** Left panel: days to death after PICU admission depicted on a histogram (top) and survival curve (bottom); n = 1,550. Right panel: months to death after PICU discharge depicted on a histogram (top) and survival curve (bottom); n = 782. Grey ribbons indicate 95% confidence intervals.


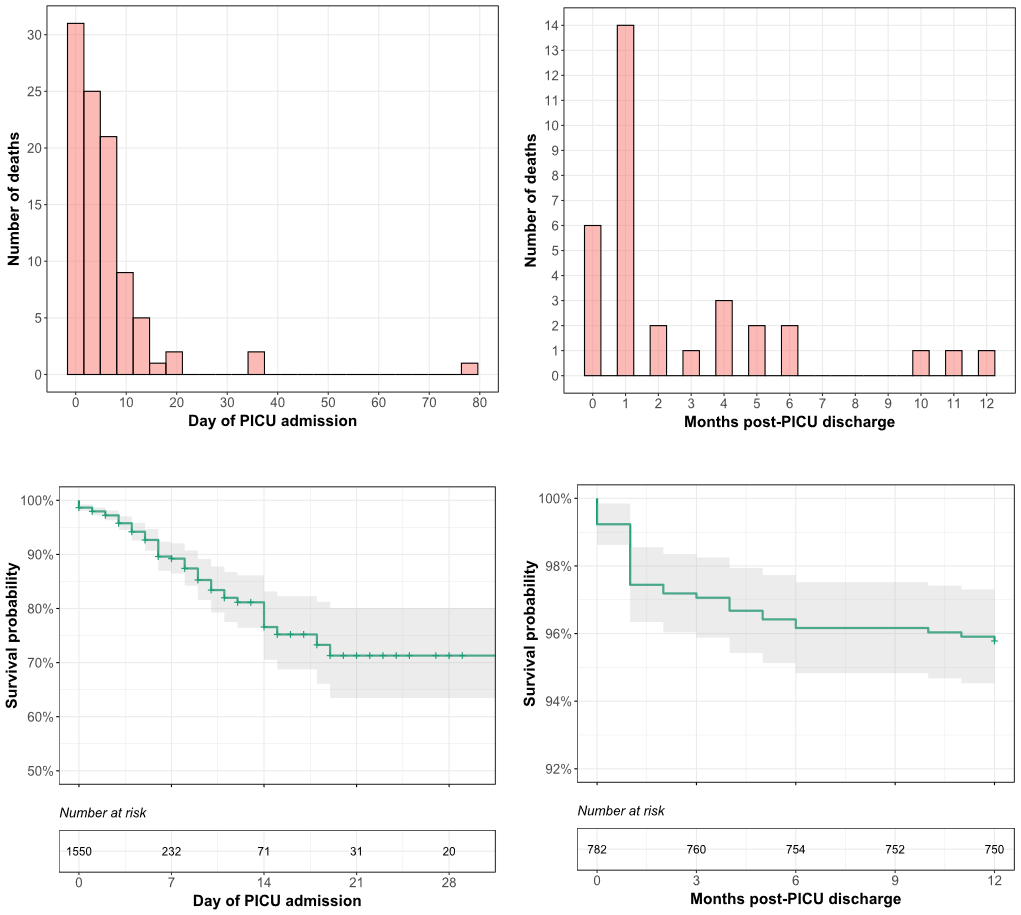


**
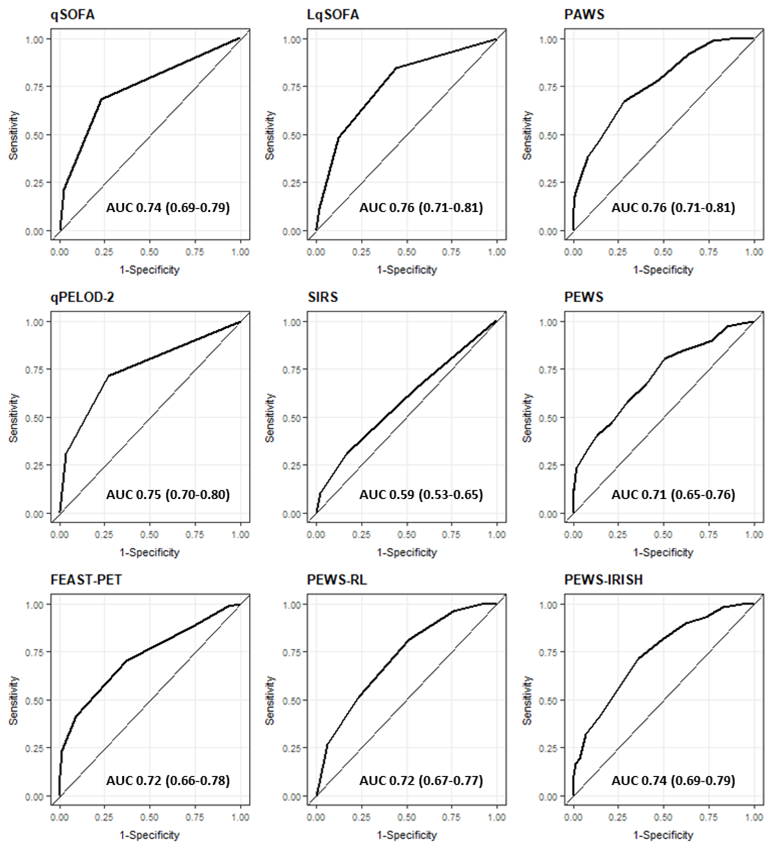
Appendix 13. Discrimination of existing severity scores.** Perfect discrimination is indicated by an AUC of 1.0. AUC = area under the receiver operating characteristic curve.

**
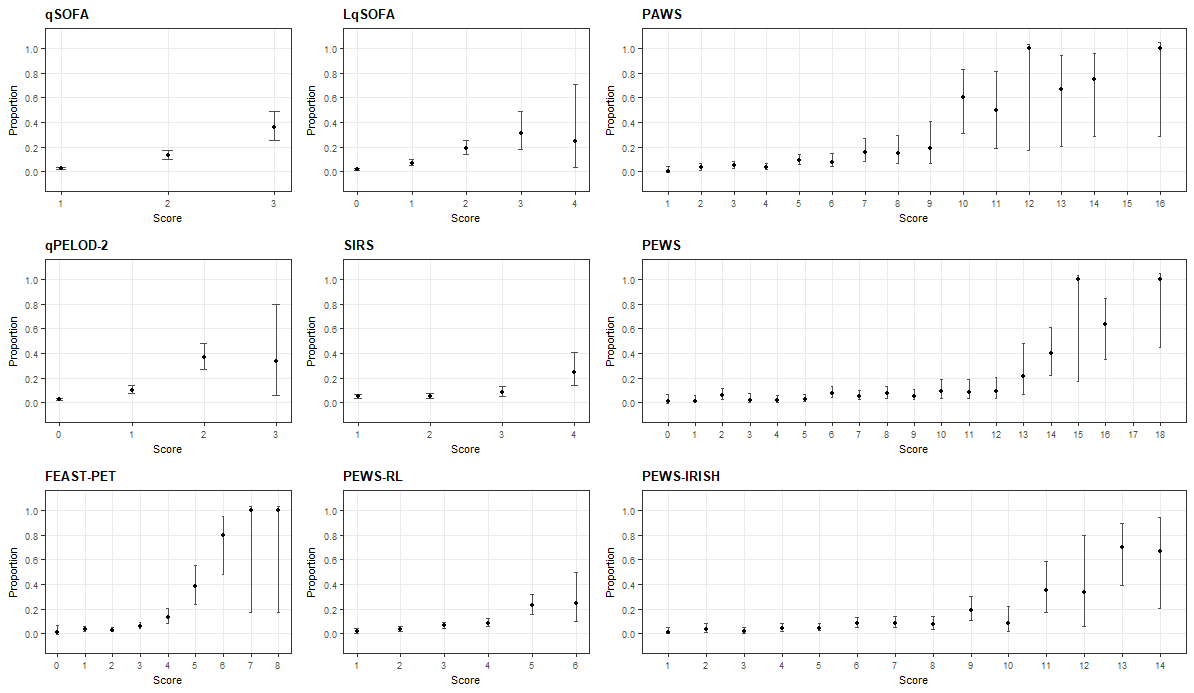
Appendix 14. Calibration of existing severity scores.** Proportion of admissions at each level of each score that died during their PICU stay. Error bars indicate Wilson 95% confidence intervals.

**Appendix 15. Sensitivity and specificity of existing severity scores.** Change in sensitivity (red line) and specificity (blue line) at increasing cut-offs of the severity scores. Grey shaded ribbons indicate 95% confidence intervals.

**
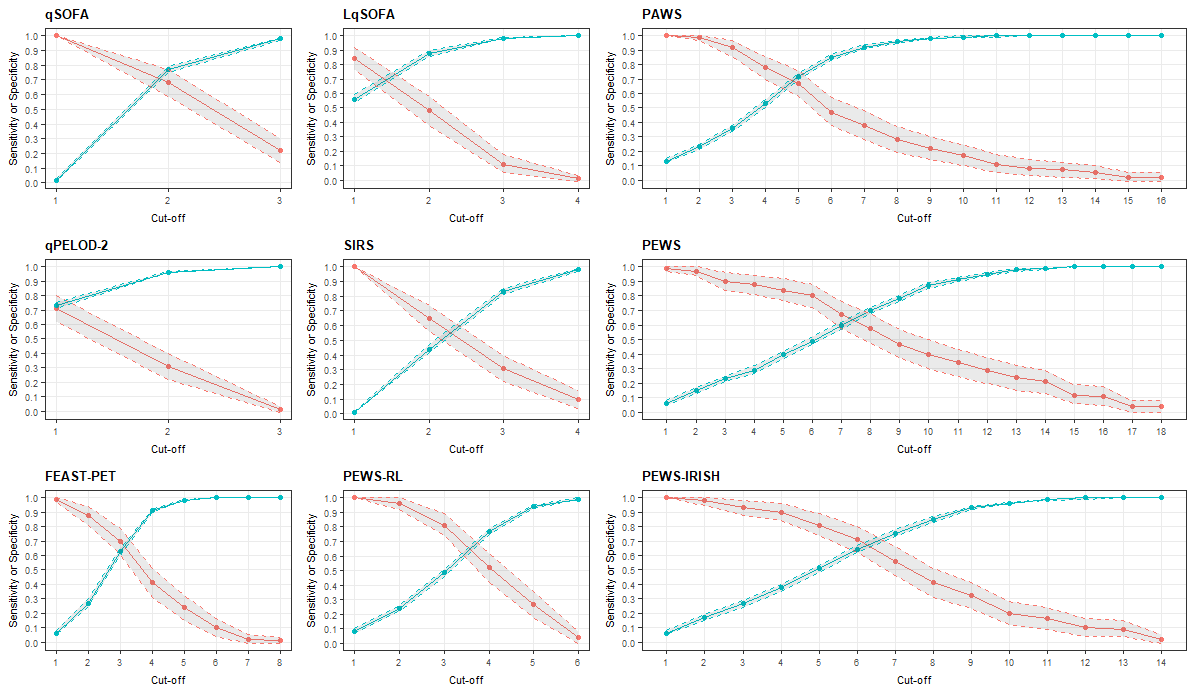
**

**Appendix 16. Negative and positive likelihood ratios of existing severity scores.** Change in negative (red line) and positive (blue line) likelihood ratios at increasing cut-offs of the severity scores, illustrated on a log_10_ scale.

**
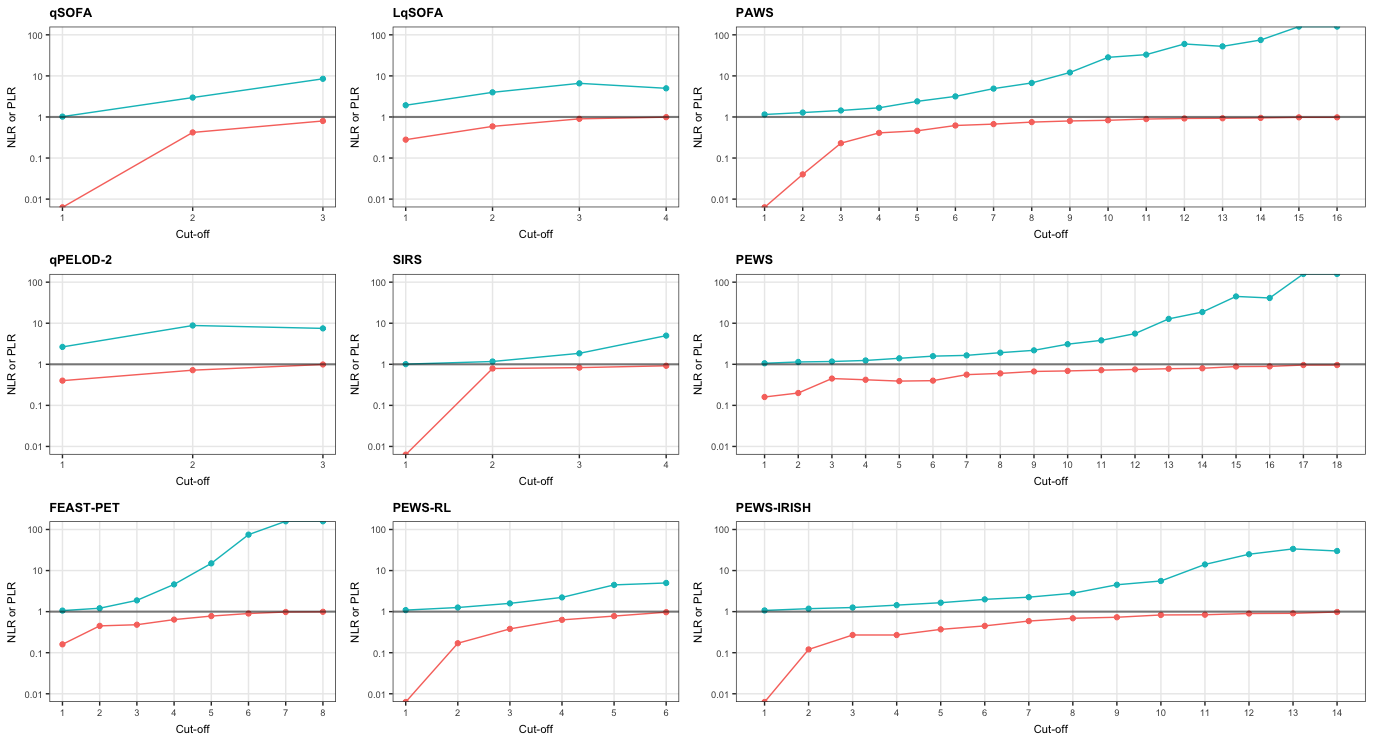
**

**
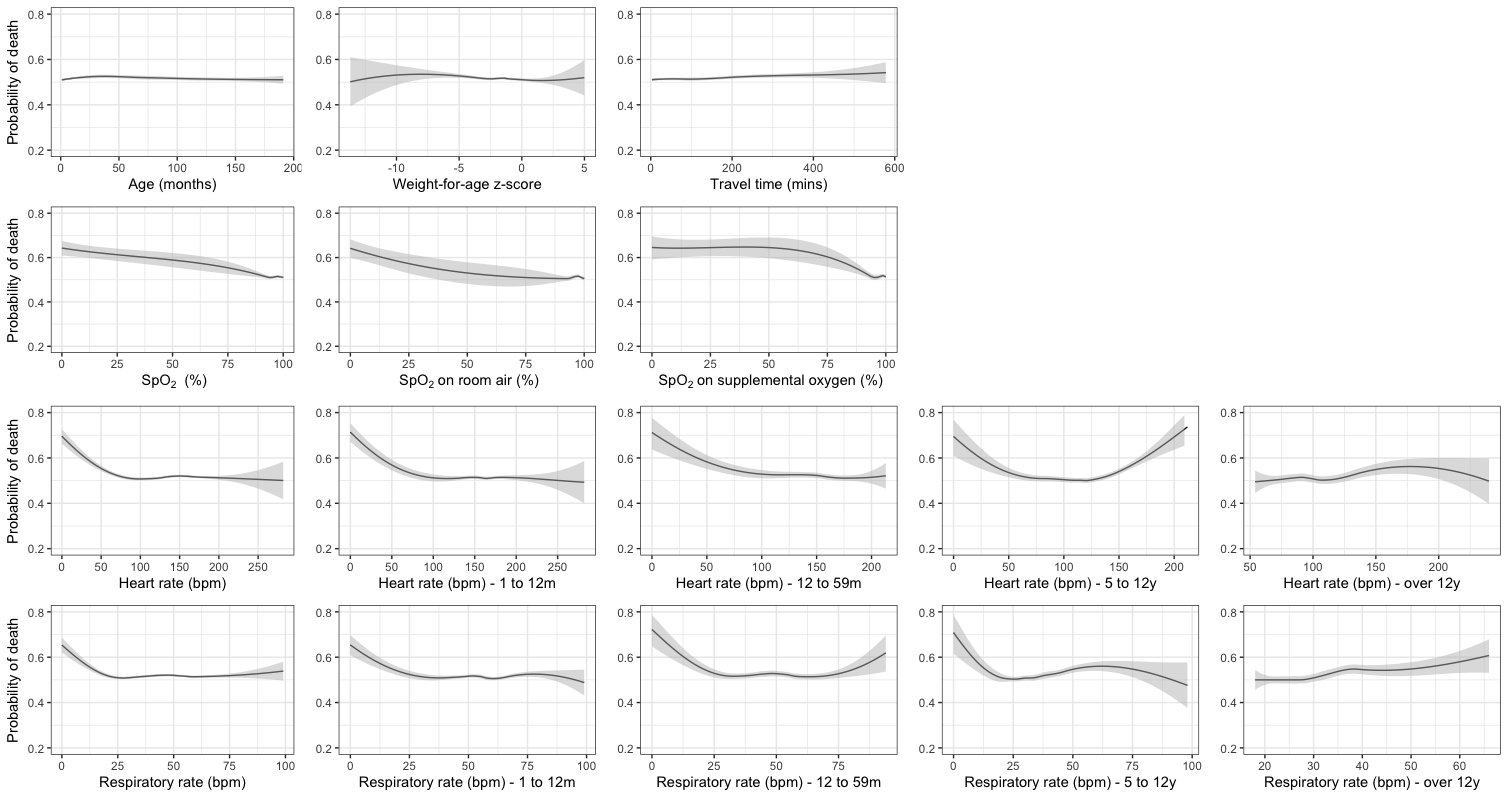
Appendix 17. Relationship between continuous candidate predictors and the primary outcome.** Locally-weighted scatterplot smoothed (LOWESS) curves to explore the relationship between continuous candidate predictors and the probability of death during PICU admission to determine if transformations might be required for the modelling. Grey ribbons indicate 95% confidence intervals.

**Appendix 18. Precision-recall curves.** Left panel = precision-recall curves of the nine existing severity scores. Right panel = precision-recall curve of the new clinical prediction model. Areas under the precision-recall curve (AUC) can be compared to a random classifier, which would have an AUC equivalent to the prevalence of the outcome, which in this case is 0.06. Precision-recall curves calculated using R package: *PRROC*.^12^


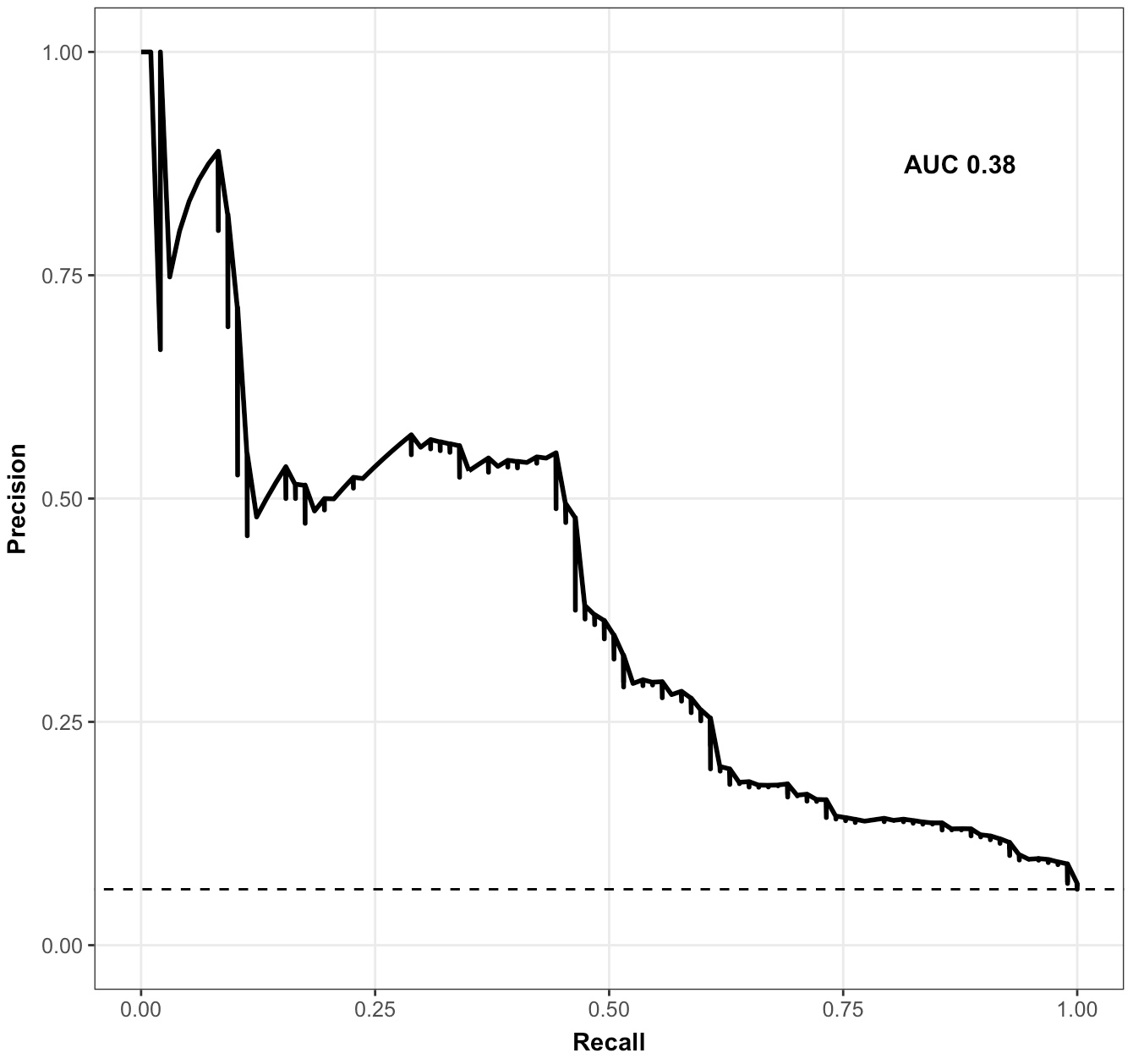

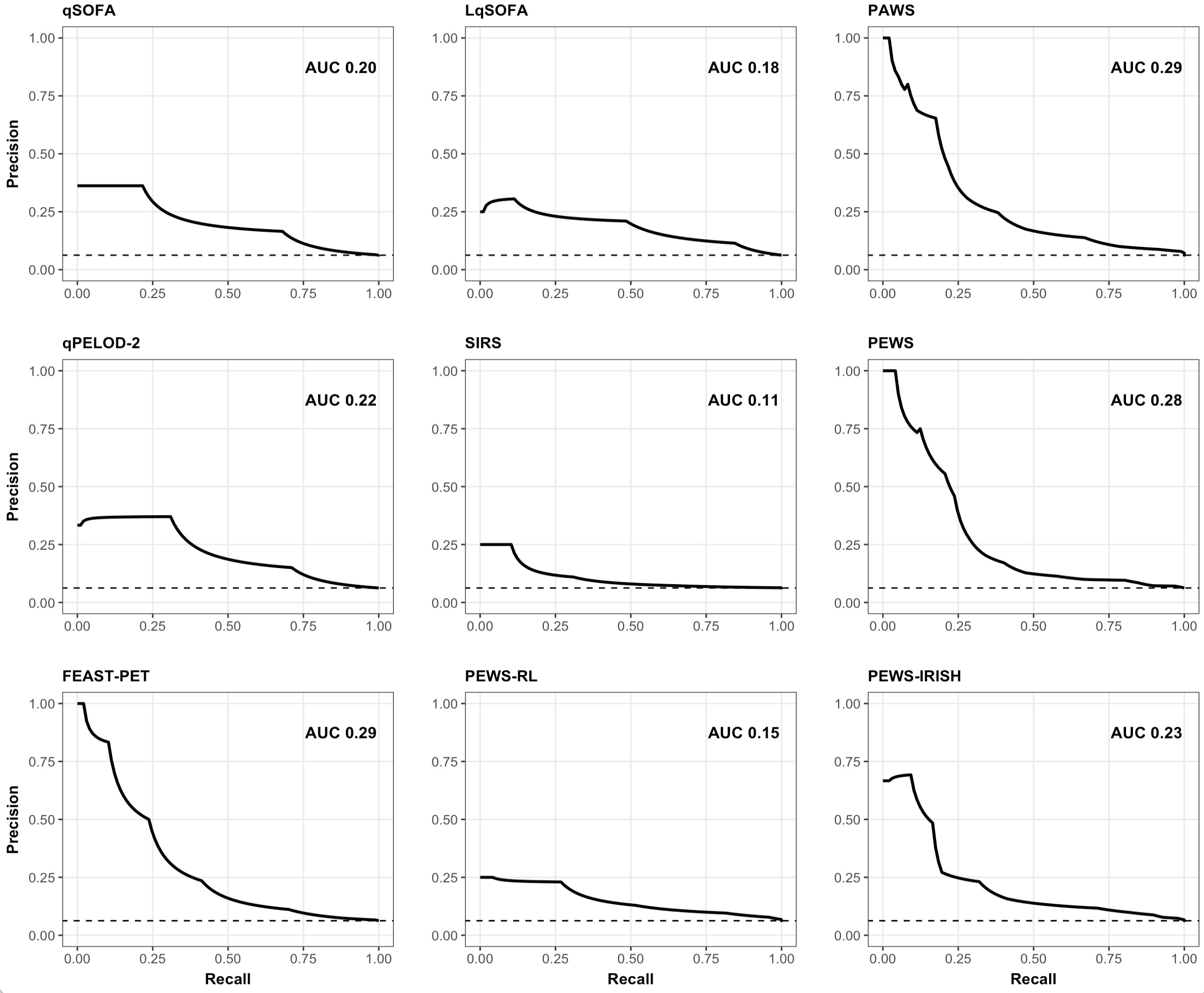


A

B

**Appendix 19. Additional classification indices illustrating ability of the model to triage PICU admissions.** Performance of the model at five cut-offs (decision thresholds or threshold probabilities). A cut-off of 10% reflects a triage strategy whereby all admissions with a predicted probability of death ≥ 10% are directed to a high-acuity area and all other admissions managed on the main unit. A decrease in threshold probability (cut-off) is associated with an increase in the sensitivity of the triage strategy for identifying high-risk admissions, at the cost of a greater proportion of admissions being directed to the high-acuity area. NLR = negative likelihood ratio; NPV = negative predictive value; PLR = positive likelihood ratio; PPV = positive predictive value.

| **Predicted probability of death** | **Specificity**  **(95% CI)** | **Sensitivity**  **(95% CI)** | **PLR**  **(95% CI)** | **NLR**  **(95% CI)** | **PPV**  **(95% CI)** | **NPV**  **(95% CI)** | **Percentage of admissions triaged as high-acuity** | **Ratio of incorrect to correct high-acuity triages** |
| --- | --- | --- | --- | --- | --- | --- | --- | --- |
| **2.5%** | 0.16  (0.14-0.18) | 0.99  (0.97-1.00) | 1.18  (1.14-1.21) | 0.07  (0.01-0.46) | 0.07  (0.06-0.09) | 1.00  (0.99-1.00) | 85.0% | 13:1 |
| **5%** | 0.63  (0.61-0.66) | 0.86  (0.79-0.93) | 2.31  (2.08-2.57) | 0.23  (0.14-0.37) | 0.13  (0.10-0.16) | 0.99  (0.98-0.99) | 40.1% | 6:1 |
| **7.5%** | 0.80  (0.78-0.82) | 0.65  (0.56-0.74) | 3.27  (2.73-3.91) | 0.44  (0.33-0.57) | 0.18  (0.14-0.22) | 0.97  (0.96-0.98) | 22.7% | 5:1 |
| **10%** | 0.90  (0.88-0.92) | 0.58  (0.48-0.68) | 5.75  (4.57-7.23) | 0.47  (0.37-0.59) | 0.28  (0.22-0.34) | 0.97  (0.96-0.98) | 13.0% | 3:1 |
| **15%** | 0.96  (0.95-0.97) | 0.46  (0.37-0.56) | 11.43  (8.22-15.88) | 0.56  (0.46-0.67) | 0.43  (0.34-0.53) | 0.96  (0.95-0.97) | 12.3% | 1:1 |


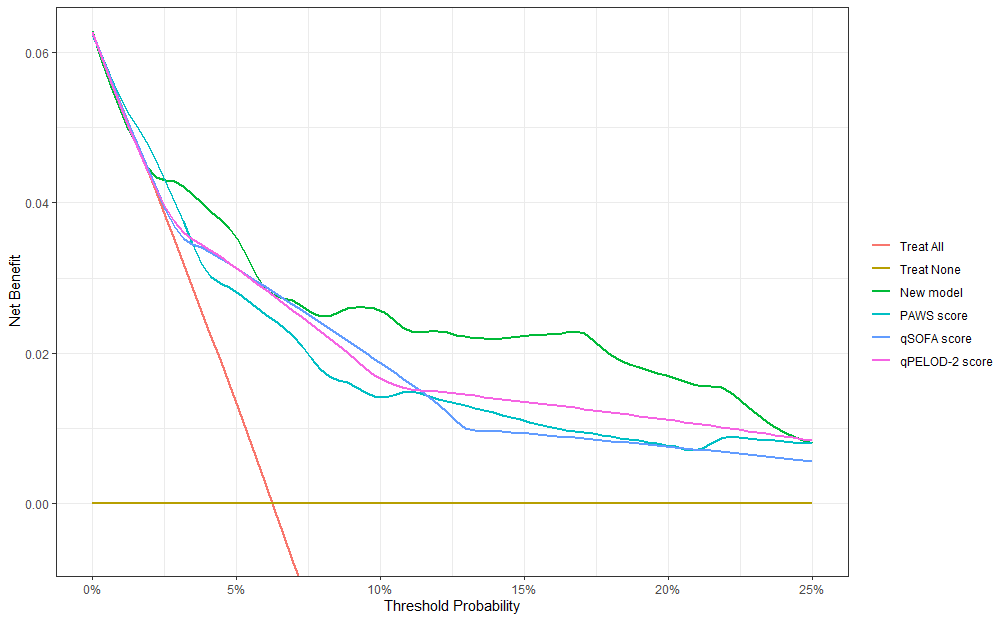
**Appendix 20. Clinical utility of the new model across a range of plausible decision thresholds (color version).** A cut-off (decision threshold or threshold probability) of 10% reflects a triage strategy whereby all admissions with a predicted probability of death ≥ 10% are directed to a high-acuity area and all other admissions managed on the main unit. The net benefit of the new model (green line) is compared to “Treat All” (red line; all PICU admissions are triaged to the high acuity area) and “Treat None” (brown line; no PICU admissions are triaged to the high acuity area) strategies, as well as the three existing scores that demonstrated potential for stratifying admissions into low- and high-risk groups from the external validation (PAWS turquoise line; qSOFA = blue line; qPELOD-2 = pink line). Above a cut-off of 7.5% using the new model to triage admissions appears to be the optimal strategy.

**REFERENCES**

1. Romaine S.T, Potter J, Khanijau A, et al. Accuracy of a Modified qSOFA Score for Predicting Critical Care Admission in Febrile Children. *Pediatrics* 2020; **146**(4): e20200782.

2. World Health Organization. Haemoglobin concentrations for the diagnosis of anaemia and assessment of severity. Vitamin and Mineral Nutrition Information System. Geneva, 2011.

3. George EC, Walker AS, Kiguli S, et al. Predicting mortality in sick African children: the FEAST Paediatric Emergency Triage (PET) Score. *BMC Med* 2015; **13**: 174.

4. Egdell P, Finlay L, Pedley DK. The PAWS score: validation of an early warning scoring system for the initial assessment of children in the emergency department. *Emerg Med J* 2008; **25**(11): 745-9.

5. Parshuram CS, Hutchison J, Middaugh K. Development and initial validation of the Bedside Paediatric Early Warning System score. *Crit Care* 2009; **13**(4): R135.

6. National Clinical Effectiveness Committee and Department of Health. National Clinical Guideline on the Irish Paediatric Early Warning System (PEWS), 2016.

7. Rosman SL, Karangwa V, Law M, Monuteaux MC, Briscoe CD, McCall N. Provisional Validation of a Pediatric Early Warning Score for Resource-Limited Settings. *Pediatrics* 2019; **143**(5): e20183657.

8. Leclerc F, Duhamel A, Deken V, Grandbastien B, Leteurtre S, Groupe Francophone de Reanimation et Urgences P. Can the Pediatric Logistic Organ Dysfunction-2 Score on Day 1 Be Used in Clinical Criteria for Sepsis in Children? *Pediatr Crit Care Med* 2017; **18**(8): 758-63.

9. Schlapbach LJ, Straney L, Bellomo R, MacLaren G, Pilcher D. Prognostic accuracy of age-adapted SOFA, SIRS, PELOD-2, and qSOFA for in-hospital mortality among children with suspected infection admitted to the intensive care unit. *Intensive Care Med* 2018; **44**(2): 179-88.

10. Goldstein B, Giroir B, Randolph A, International Consensus Conference on Pediatric S. International pediatric sepsis consensus conference: definitions for sepsis and organ dysfunction in pediatrics. *Pediatr Crit Care Med* 2005; **6**(1): 2-8.

11. Pollack MM, Dean JM, Butler J, et al. The ideal time interval for critical care severity-of-illness assessment. *Pediatr Crit Care Med* 2013; **14**(5): 448-53.

12. Keilwagen J, Grosse I, Grau J. Area under Precision-Recall Curves for Weighted and Unweighted Data. *PLoS One* 2014; **9**: e92209.
